# Step-Growth Glycopolymers with a Defined Tacticity for Selective Carbohydrate–Lectin Recognition

**DOI:** 10.1021/acs.biomac.3c00133

**Published:** 2023-03-28

**Authors:** Jonas Becker, Roberto Terracciano, Gokhan Yilmaz, Richard Napier, C. Remzi Becer

**Affiliations:** † Department of Chemistry, University of Warwick, Coventry CV4 7AL, U.K.; ‡ School of Life Sciences, University of Warwick, Coventry CV4 7AL, U.K.

## Abstract

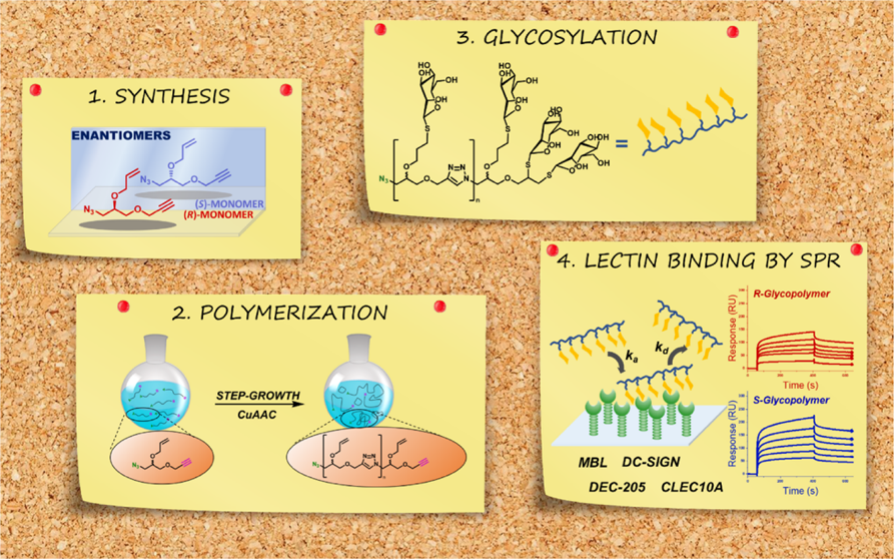

Glycopolymers are potent candidates for biomedical applications by exploiting multivalent carbohydrate–lectin interactions. Owing to their specific recognition capabilities, glycosylated polymers can be utilized for targeted drug delivery to certain cell types bearing the corresponding lectin receptors. A fundamental challenge in glycopolymer research, however, is the specificity of recognition to receptors binding to the same sugar unit (e.g., mannose). Variation of polymer backbone chirality has emerged as an effective method to distinguish between lectins on a molecular level. Herein, we present a facile route toward producing glycopolymers with a defined tacticity based on a step-growth polymerization technique using click chemistry. A set of polymers have been fabricated and further functionalized with mannose moieties to enable lectin binding to receptors relevant to the immune system (mannose-binding lectin, dendritic cell-specific intercellular adhesion molecule-3-grabbing non-integrin, and dendritic and thymic epithelial cell-205). Surface plasmon resonance spectrometry was employed to determine the kinetic parameters of the step-growth glycopolymers. The results highlight the importance of structural complexity in advancing glycopolymer synthesis, yet multivalency remains a main driving force in lectin recognition.

## Introduction

Over the last few decades, glycobiology has emerged into one of the key fields of research for the development of novel therapeutics.^1−4^ Carbohydrates provide specific interactions in cellular recognition events, in which lectins play a fundamental role.^5−7^ Biologically, lectins are involved in inflammation,^8^ virus onset,^9−11^ and cell–cell communication.^12^ Therefore, they bear a strong potential as drug targets.^13−15^

Glycopolymers have proven to be a powerful tool for the investigation of carbohydrate–lectin interactions.^16−18^ Owing to the multivalent structure, these polymers with pendant carbohydrates exhibit strong interactions with their lectin counterparts.^19−21^ Notably, the affinity of binding can be tailored depending on the polymer length,^22,23^ architecture,^24−26^ rigidity,^27^ and nature of carbohydrate. A crucial challenge, however, remains—selectivity toward specific lectins.^28−30^ Within a cohort of lectins binding to the same sugar unit, the structure of the polymer backbone is decisive over the magnitude of interaction with the individual lectins. Hence, new levels of complexity are desired to fabricate glycopolymers with the ability to distinguish between lectins.

One way to differentiate between lectins has been presented by Johnson et al., utilizing chirality as a measure to control the interaction between carbohydrate ligands and mannose-binding lectins (MBLs).^31^ It has been shown that the absolute stereoconfiguration of glycooligomers derived by iterative exponential growth (IEG) substantially influences the binding behavior, enabling selective interactions toward human lectins.

Step-growth polymerization is one of the oldest techniques in the synthesis of polymeric materials known to scientists.^32^ It is used in a plethora of applications in materials science, as it represents a facile way of making polymers by the reaction of at least two bifunctional monomers with the same functional group on either end (A–A; B–B) or the polymerization of a bifunctional monomer with different functional groups at the α- and ω-ends (A–B). Performing step-growth polymerization based on “click” reactions such as the copper-catalyzed azide alkyne cycloaddition (CuAAC) renders a very efficient way to synthesize polymers in high yields and in a variety of reaction media.^33−36^

In this study, we have investigated the effect of stereoconfiguration of step-growth-derived glycopolymers on lectin binding. It was hypothesized that CuAAC-derived polytriazoles obtained from step-growth polymerization are synthetically easier to access than monodisperse oligomers with a discrete sequence. Additionally, we presumed that the selectivity induced by the chirality of the backbone would result in the ability to target specific lectins. A set of new A–B-type monomers with different chiralities were synthesized, and their performance in step-growth polymerization as well as post-functionalization with mannose units by thiol–ene chemistry was investigated. The lectin-binding properties of the obtained glycomaterials were analyzed by surface plasmon resonance (SPR) with a group of biologically relevant MBLs [dendritic cell-specific intercellular adhesion molecule-3-grabbing non-integrin (DC-SIGN), MBL, and dendritic and thymic epithelial cell-205 (DEC-205)].^37^ All three lectins have natural functions in the immune system, providing potential targets for specific drug or vaccine delivery.^38−41^ Additionally, the affinity to a galactosamine-specific lectin CLEC10A (aka MGL)^42^ was tested to elucidate any nonspecific interactions.

## Experimental Section

### Materials and Methods

2-Mercaptoethanol (≥99.0%, Sigma-Aldrich), acetonitrile (MeCN, 99.9%, extra dry, Acros Organics), allyl bromide (99%, stabilized, Acros Organics), azobisisobutyronitrile (AIBN, 98%, Sigma-Aldrich), boron trifluoride etherate (for synthesis, Sigma-Aldrich), copper(I) bromide (Sigma-Aldrich), dimethylformamide (DMF, 99.8%, extra dry, Thermo Fisher Scientific), epichlorohydrin (99%, Acros Organics), *R*-(−)-epichlorohydrin (99%, Acros Organics), *S*-(+)-epichlorohydrin (98%, Sigma-Aldrich), sodium azide (purum, ≥99.0%, Sigma-Aldrich), sodium hydride (60% dispersion, mineral oil), sodium methoxide (reagent grade, 95%, Sigma-Aldrich) were purchased from commercial suppliers and used as received. All moisture-sensitive reactions were carried out under an inert atmosphere of nitrogen using standard syringe/septa techniques.

Nuclear magnetic resonance (NMR) spectroscopy was performed on a Bruker Avance III HD 300 MHz or Bruker Avance III HD 400 MHz instrument. Deuterated solvents were used, and the signal of the residual solvent served as a reference for the chemical shift, δ.

Gel permeation chromatography (GPC) measurements in tetrahydrofuran (THF) were performed on an Agilent Technologies 1260 Infinity using THF as the eluent with 2% triethylamine. The instrument was equipped with a refractive index (RI) and a ultraviolet (UV) detector at 308 nm, a PLgel 5 μm guard column, and a PLgel 5 μm mixed D column (300 × 7.5 mm). Samples were run at 1 mL min^–1^ at 40 °C. Poly(methyl methacrylate) standards (Agilent PMMA calibration kits, M-M-10 and M-L-10) were used for the calibration. Before injection (100 μL), the samples were filtered through a PTFE membrane with a 0.2 μm pore size. Experimental molar mass (*M*_n_), weight-average molar mass (*M*_w_), and dispersity (*D̵*) values of synthesized polymers were determined by conventional calibration using Agilent GPC/SEC software.

GPC measurements in DMF were carried out on an Agilent 1260 Infinity II-MDS instrument with two PLgel mixed-D columns operating in DMF with 5 mM NH_4_BF_4_ and equipped with the following detectors: RI, viscometer, light scattering (LS), and variable wavelength detector (VWD). The instrument was calibrated with linear poly(methyl methacrylate) standards (500 Da–1500 kDa). All samples were passed through 0.2 μm nylon filters prior to GPC measurements.

Chiral high-performance liquid chromatography (HPLC) was performed on an Agilent 1260 Infinity II system equipped with a CHIRALPAK IA column (4.6 mm ID, 250 mmL, 5 μm) using a mixture of hexanes/isopropanol 2% and a UV–vis detector.

Circular dichroism (CD) spectra were acquired on a Jasco J-1500 spectrometer at 25 °C using a 1 mm cuvette. Sample solutions were prepared at a concentration of 1.0 mg/mL and filtered through a 0.2 μm nylon filter. If the CD scan showed a high tension voltage (HT) of over 700 V, the sample was diluted down to stay below that threshold.

Matrix-assisted laser desorption ionization–time-of-flight mass spectrometry (MALDI–ToF MS) was performed on a Bruker Daltonics Autoflex spectrometer equipped with a nitrogen laser at 337 nm with positive ion detection. Polymer samples were prepared as follows: solutions in THF of *trans*-2-[3-(4-*tert*-butylphenyl)-2-methyl-2-propenylidene]malononitrile (DCTB, ≥98%) as matrix (20 mg/mL), sodium trifluoroacetate (NaTFA) as cationization agent (10 mg/mL), and sample (5 mg/mL) were mixed in a ratio of 5/2/5 and then spotted onto the target (0.5 μL). Spectra were recorded in reflective mode, and the mass spectrometer was calibrated with a PMMA standard up to 3 kDa.

SPR was used for interaction analysis for all lectins. The extent of interaction between the glycopolymers and lectins was analyzed on a BIAcore T200 system (Cytiva Life Sciences). The lectins (0.005 mg/mL) were immobilized via a standard amino coupling protocol onto a CM5 sensor chip that was activated by flowing a 1:1 mixture of 0.1 M *N*-hydroxysuccinimide and 0.05 M *N*-ethyl-*N*′ (dimethylaminopropyl)carbodiimide over the chip for 5 min at 25 °C at a flow rate of 5 μL/min after the system equilibration with HEPES filtered buffer (10 mM HEPES pH 7.4, 150 mM NaCl, 5 mM CaCl_2_). Subsequently, channels 1 (blank), 2, 3, and 4 were blocked by flowing a solution of ethanolamine (1 M pH 8.5) for 10 min at 5 μL/min to block the remaining reactive groups on the channels. Sample solutions were prepared at varying concentrations (8.0–0.25 μM) in the same HEPES buffer to calculate the binding kinetics. Sensorgrams for each glycopolymer concentration were recorded with a 350 s injection of polymer solution (on period), followed by 200 s of buffer alone (off period). Regeneration of the sensor chip surfaces was performed using 10 mM HEPES pH 7.4, 150 mM NaCl, 10 mM EDTA, and 0.01% Tween 20 surfactant solution. Kinetic data were evaluated using a single set of sites (1:1 Langmuir binding) model in the BIAevaluation 3.1 software.

### General Procedure for the Step-Growth Polymerization of Monomers **2**, **2*R***, and **2*S***

Under an N_2_ atmosphere, monomer **2** (0.50 g, 2.55 mmol) and PMDETA (0.05 mL, 0.25 mmol) were dissolved in anhydrous THF or DMF (2.2 mL) in an oven-dried Schlenk tube. The mixture was heated to 45 °C and degassed for 15 min. Then, CuBr (0.019 g, 0.13 mmol) was added, and the mixture was stirred for 4 h. The mixture was precipitated into cold diethyl ether (200 mL) and filtered. The residue was dissolved in DCM (20 mL) and washed with H_2_O (3 × 20 mL). The organic phase was dried over MgSO_4_, filtered, and the solvent removed under reduced pressure. Polymer **3** was obtained as an amber solid (0.302 g, 60%).

^1^H NMR (400 MHz, CDCl_3_): δ (ppm) 7.68 (s, 1H, H^Ar^), 5.71 (m, 1H, CH=CH_2_), 5.14 (m, 2H, CH=CH_2_), 4.66 (s, 2H, OCH_2_Ar), 4.58 (m, 1H, CHCH_2_O), 4.43 (m, 1H, CHCH_2_O), 4.03 (m, 1H, OCH_2_C=C), 3.89 (m, 2H, OCH_2_C=C + CH_2_CHOCH_2_), 3.54 (m, 2H, ArCH_2_CH).

### General Procedure for Thiol–ene Glycosylation of Allyl Polytriazoles **3**, **3*R***, and **3*S***

Under an N_2_ atmosphere, **3** (0.092 g) was dissolved in anhydrous acetonitrile (1 mL). Ac_4_Man-SH (1.03 g, 2.82 mmol, 6.00 equiv per ene) and AIBN (0.02 g, 0.12 mmol, 0.25 equiv per ene) were added, and the suspension was degassed for 15 min. The mixture was stirred at 70 °C for 18 h and then poured into cold diethyl ether (200 mL) and filtered. The residue was collected and dried under reduced pressure. The obtained crude intermediate product was dissolved in MeOH (10 mL), sodium methoxide (0.04 g, 0.71 mmol, 1.5 equiv per mannose unit) was added, and the mixture was stirred at ambient temperature. After 18 h, the solvent was removed under reduced pressure and the crude product was dissolved in H_2_O (6 mL). The solution was transferred into a dialysis bag (MWCO: 1 kDa) and dialyzed against H_2_O for 2 days. Lyophilization afforded the glycopolymer product **4** (0.041 g, 22%) as a white solid.

^1^H NMR (400 MHz, D_2_O): δ (ppm) 8.08 (s, 1H, H^Ar^), 5.01–4.44 (m, 3H, OCH_2_CAr + H^1^ + CHCH_2_O), 4.13–3.94 (m, 2H, OCHCH_2_CH_2_), 3.93–3.81 (m, 2H, CH_2_CHOCH_2_ + H^2^), 3.77–3.50 (m, 6H, H^3^ + H^4^ + H^5^ + H^6^), 3.49–3.24 (m, 2H, NCH_2_CH), 2.67–2.34 (m, 2H, SCH_2_CH_2_), 1.84–1.58 (m, 2H, CH_2_CH_2_CH_2_).

## Results and Discussion

### Monomer Synthesis

**Scheme 1:**
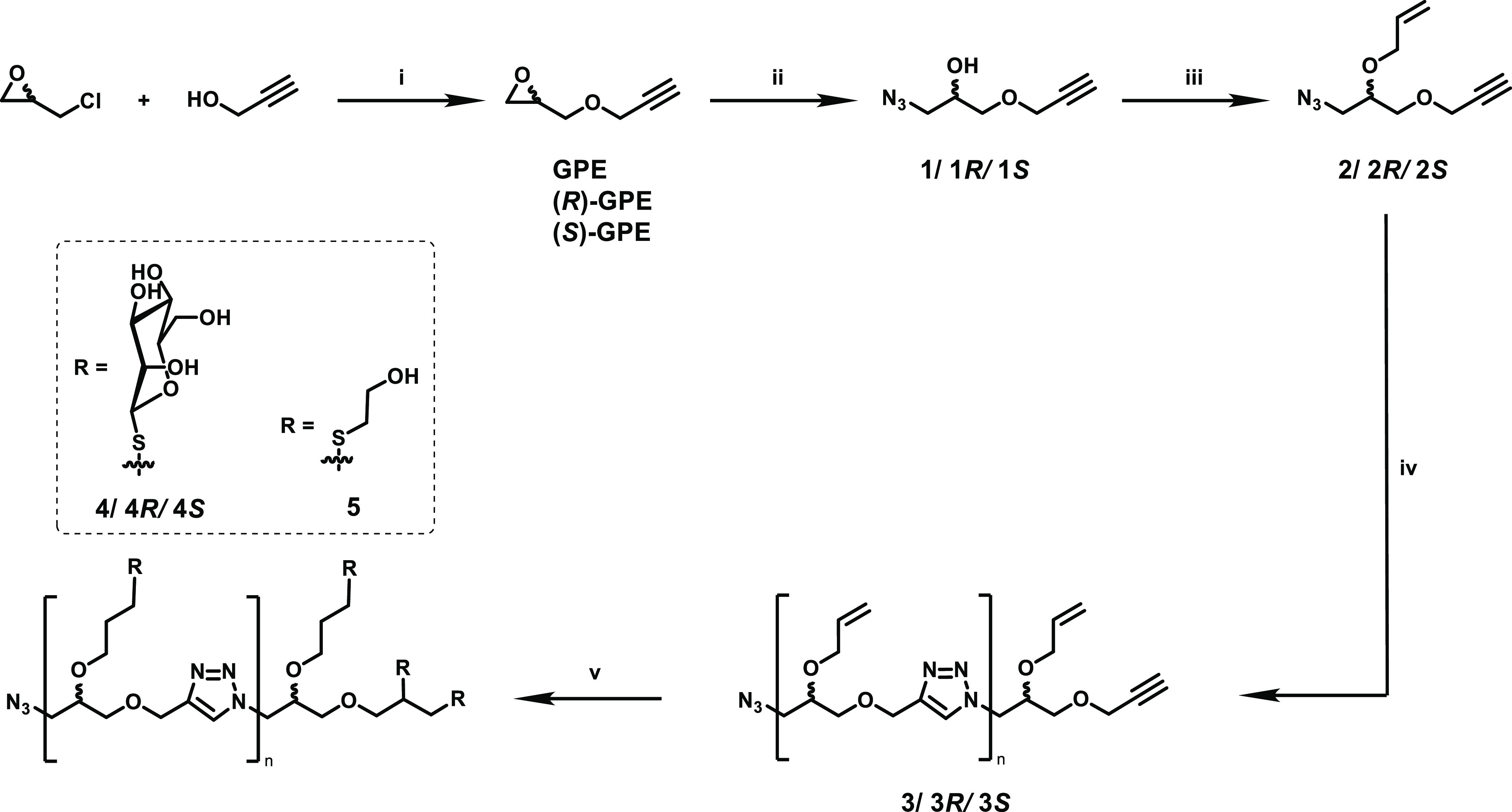
Synthesis of Step-Growth Glycopolymers **4**, **4*R***, and **4*S*** and Reference Polymer **5** (i) **1**, BF_3_(OEt)_2_, 0 °C to r.t., 2 h/**2**, 25% NaOH (aq), r.t., 15 min. (ii) NaN_3_, AcOH, DMF, 70 °C, 18 h. (iii) Allyl bromide, NaH, DMF, 0 °C to r.t., 18 h. (iv) CuBr, PMDETA, DMF, 45 °C, 4 h. (v) **1**, Ac_4_ManSH or 2-mercaptoethanol, AIBN, MeCN, 70 °C, 18 h/**2**, NaOMe (cat.), MeOH, r.t., 18.

Azide–alkyne step-growth monomers were prepared from a modified method based on the work by Johnson and co-workers^43,44^ in a three-step synthesis starting from epichlorohydrin (Scheme 1). Epoxide ring-opening with propargyl alcohol and subsequent elimination to reform the ring afforded glycidyl propargyl ether (GPE). Treatment with excess sodium azide gave rise to secondary alcohols **1**, **1*R***, and **1*S***. Reaction time was limited to 4 h in this step to minimize potential thermal polymerization. Ultimately, ether formation with allyl bromide was performed to yield the desired monomers **2**, **2*R***, and **2*S***.

**Figure 1:**
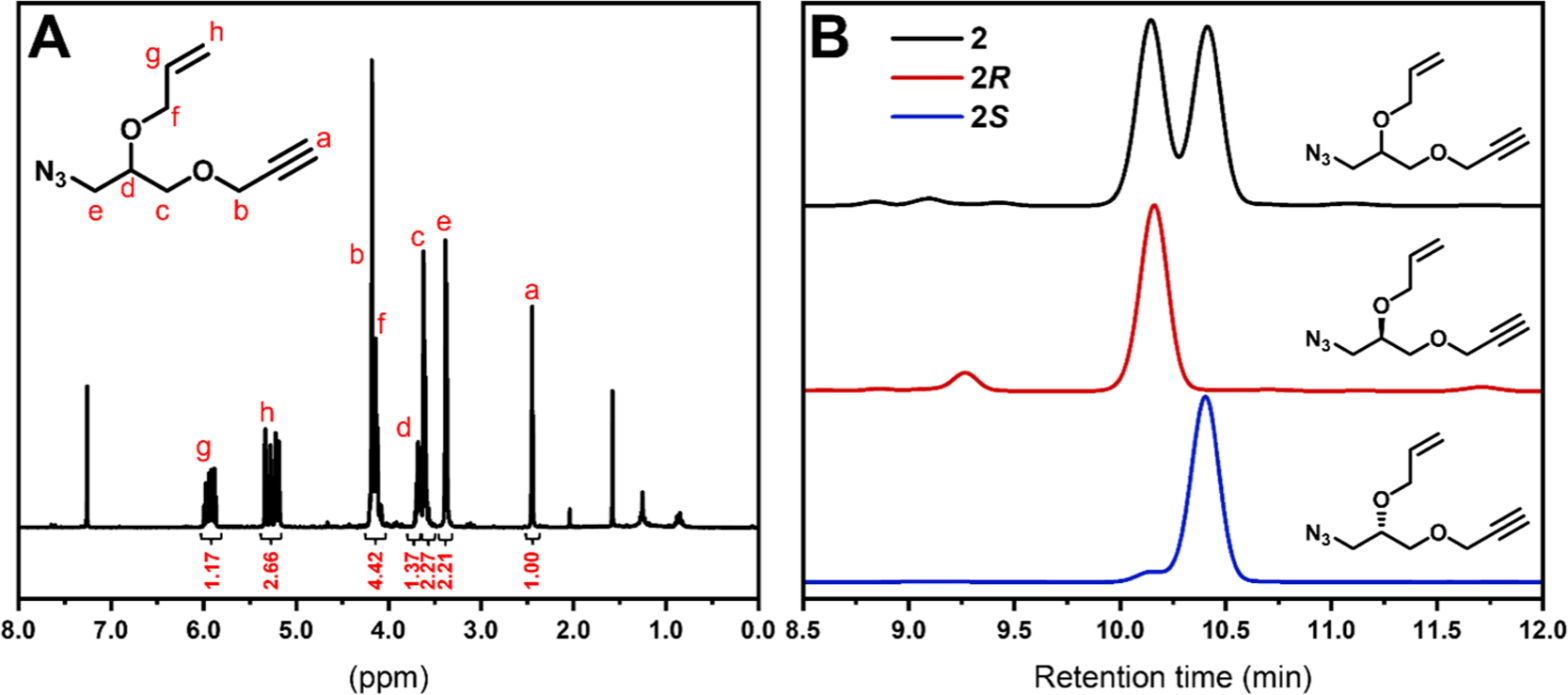
(A) ^1^H NMR spectrum of **2** (CDCl_3_, 400 MHz). (B) Chiral HPLC traces of **2**, **2*R***, and **2*S*** (column: CHIRALPAK IA, mobile phase *n*-hexane/IPA 2%, 31 °C, 0.5 mL/min, λ_detection_ = 220 nm).

To confirm the chiral purity, the new monomers were analyzed by chiral HPLC (Figure 1). The measurements were carried out in normal phase conditions consisting of *n*-hexane/IPA 2%. The chiral HPLC trace of the allyl monomer racemate **2** showed two not fully separated peaks at 10.15 and 10.42 min, respectively. These were assigned to the two enantiomers present in the mixture when compared to the traces of the chiral monomers **2*R*** and **2*S*** obtained from enantiopure precursors. Both traces showed only one main peak at the corresponding retention times, indicating that the synthetic procedures lead to satisfactory enantiomeric purity. Notably, the chiral HPLC trace for **2*S*** showed a small residue peak of the opposite isomer present in the mixture. For **2*R***, however, no detectable *S*-enantiomer in the region of interest was observed, suggesting that the polymers derived from these new monomers can exhibit differences in physical properties depending on their stereochemistry.

### Step-Growth Polymerization via CuAAC

Monomer **2** was polymerized in the presence of a catalytic system consisting of CuBr and PMDETA at 5 and 10 mol %, respectively. Polymerization conditions were screened, and polymers **3a–3f** were analyzed by GPC (Table 1). Initially, THF was used as the reaction solvent with a monomer concentration of 100 mg/mL. GPC analysis revealed a *M*_n_-value of 2600 Da and a relatively broad dispersity of 1.92 after 18 h at 45 °C. Additionally, the GPC trace showed a small molecular weight fraction at 10.1 min (Supporting Information, Figure S4), indicating the formation of a cyclic byproduct, which is inherent to step-growth polymerizations. Consequently, the monomer concentration was increased to 225 mg/mL to reduce the emergence of cyclic byproduct. As expected, the peak in the small molecular weight region decreased at higher concentrations. The molecular weight of the main peak, however, indicated slightly lower *M*_n_ and *M*_w_. The polymerization was monitored over several points in time, and it was found that the polymer peak in the GPC traces is present after 15 min and does not change significantly over the course of the reaction up to 18 h (Supporting Information, Figure S3). Hence, for the remaining experiments, the reaction time was shortened to 4 h. We presumed that a more polar reaction solvent could lead to an increase in the molecular weight of the polytriazoles due to the polar nature of the triazole moiety. Therefore, DMF was utilized in **3d** instead of THF, however, no shift to higher molecular weight in the main polymer peak was observed (Supporting Information, Figure S5). Additionally, the concentration of catalyst was altered in **3e** and **3f** to 10 and 15 mol %, respectively. Likewise, the amount of PMDETA was adjusted to match the Cu(I) content (Supporting Information, Figure S6). A higher catalyst content was assumed to increase the overall molecular weight of the resulting polymer due to prolonged elongation of the chains. Consequently, a slight increase in molecular weight was observed for both polymers, while their dispersity remained in a similar range. Overall, the findings from this screening of conditions underpin the advantages of CuAAC-based polymerization, proceeding efficiently and providing polytriazoles with satisfactory characteristics to continue to fabricate chiral glycopolymers. However, from the iterations of reaction conditions performed, no substantial effect on the overall molecular weight was observed. Therefore, it was noted that monomer purity is the key factor governing the average DP of this step-growth reaction.

**Table 1:** GPC Results of CuAAC-Based Step-Growth Polymerization of **2** to Obtain Polytriazole **3** in Various Reaction Conditions (GPC Solvent: THF; for GPC Traces, See Supporting Information, Figures S4–S6)

polymer	monomer conc. [mg/mL]	reaction solvent	reaction time [h]	temp. [°C]	catalyst conc (CuBr/PMDETA) [mol %]	*M*_n,GPC_ [Da]	*M*_w,GPC_ [Da]	*D̵*
**3a**	100	THF	18	45	5/10	2600	5000	1.92
**3b**	225	THF	18	45	5/10	2300	3700	1.61
**3c**	225	THF	4	45	5/10	2300	3800	1.65
**3d**	225	DMF	4	45	5/10	2100	3200	1.52
**3e**	225	DMF	4	45	10/10	2600	4000	1.54
**3f**	225	DMF	4	45	15/15	2400	3700	1.54

Prior to making target glycopolymers, the highest theoretical DP from the novel monomers has been calculated. To achieve this, the end group purity of monomers **2**, **2*R***, and **2*S*** was analyzed by ^1^H NMR, that is, the integrals of the alkyne proton at 2.45 ppm and the methylene group adjacent to the azide functionality at 3.38 ppm were used as a measure to determine the stoichiometric imbalance of end groups. From these values, Carothers’s equation (Supporting Information, equation I) was employed to calculate the maximal possible average DP, where *r* indicates the stoichiometric imbalance and *p* indicates the monomer conversion.^45^ In the case of 100% monomer conversion, *p* = 1 and Carothers’s equation I reduces to II. The stoichiometric imbalance is determined for a A–B bifunctional monomer with a monofunctional impurity B′ (Supporting Information, equation III), with *N*_X_ standing for the number of end groups present in the reaction. The maximal possible average DPs for all monomers range from 20 to 79 (Supporting Information, Table S1).

In order to fabricate step-growth glycopolymers, monomers **2**, **2*R***, and **2*S*** were polymerized in the conditions used for **3e**. The resulting polytriazoles **3**, **3*R***, and **3*S*** were purified by precipitation into diethyl ether and subsequent aqueous work-up to remove the copper catalyst. After precipitation, the content of the low molecular weight byproduct was reduced significantly as observed by GPC (Supporting Information Figure S7). Furthermore, the formation of the polytriazoles can be confirmed by the emerging signal for the triazole proton at 7.68 ppm. GPC analysis was carried out on two different instruments using either THF or DMF as the elution solvent (Table 2). It can be observed that the RI traces as well as the calculated molecular weight differ greatly between both instruments (Supporting Information, Figures S9 and S11). A similar trend in DP between the three step-growth polymers is determined on both GPCs. **3*S*** was found to exhibit the highest *M*_n_ values in both measurements. Conversely, polymer **3** obtained from the racemic monomer has the lowest DP and molecular weight on the THF instrument, while the data from the DMF instrument indicates a slightly higher *M*_n_ than **3*R***. Generally, data obtained from GPC in DMF seemed to give higher values for calculated DP (derived from experimental *M*_n_ and repeat unit molar mass) and higher dispersities. MALDI–ToF mass spectrometry (Supporting Information, Figure S13) underpins the trend in molecular weight of the step-growth polytriazoles, with **3** showing signals corresponding to shorter chains, while for **3*S***, signals up to 5 kDa are observed. Moreover, the DP was calculated from NMR by integration of the triazole peak of the polymer chain at 7.66 ppm and the alkyne proton at 2.48 ppm. The NMR values indicate a DP of 19 for **3**, 29 for **3*R***, and 42 for **3*S***, where the latter two are in good agreement with the DP calculated from the DMF GPC measurements. When compared to the calculated values for DP_max_, the experimental data matches the prediction that racemic polymer **3** shows the lowest molecular weight, although the trend for a higher DP in **3*R*** than **3*S*** was found to be reversed. It must be mentioned that the calculation from Carothers’ equation also takes into consideration the DP of the cyclic product, which has been removed from the final polymers.

**Table 2:** GPC Results of Polytriazoles before Carbohydrate Addition (**3**, **3*R***, and **3*S***), Final Glycopolymers (**4**, **4*R***, and **4*S***) after Purification, and Reference Polymer **5**

polymer	stereo-config	GPC eluent	*M*_n,GPC_ [Da]	*M*_w,GPC_ [Da]	*D̵*	DP_calc.,GPC_	DP_calc.,NMR_
**3**	Rac	THF	2200	3100	1.41	11	19
**3*R***	R	THF	3600	5200	1.44	18	29
**3*S***	S	THF	4300	7400	1.72	22	42
**3**	Rac	DMF	6700	11,500	1.72	34	19
**3*R***	R	DMF	6000	9400	1.57	31	29
**3*S***	S	DMF	8500	18000	2.12	44	42
**4**	Rac	DMF	5200	9700	1.87	13	n.d.
**4*R***	R	DMF	12,000	18,300	1.53	31	n.d.
**4*S***	S	DMF	12,100	20,700	1.71	31	n.d.
**5**	Rac	DMF	9300	33,300	3.58	34	n.d.

### Thiol–Ene Addition of Thiomannose to Allyl-Containing Polytriazoles

**Figure 2:**
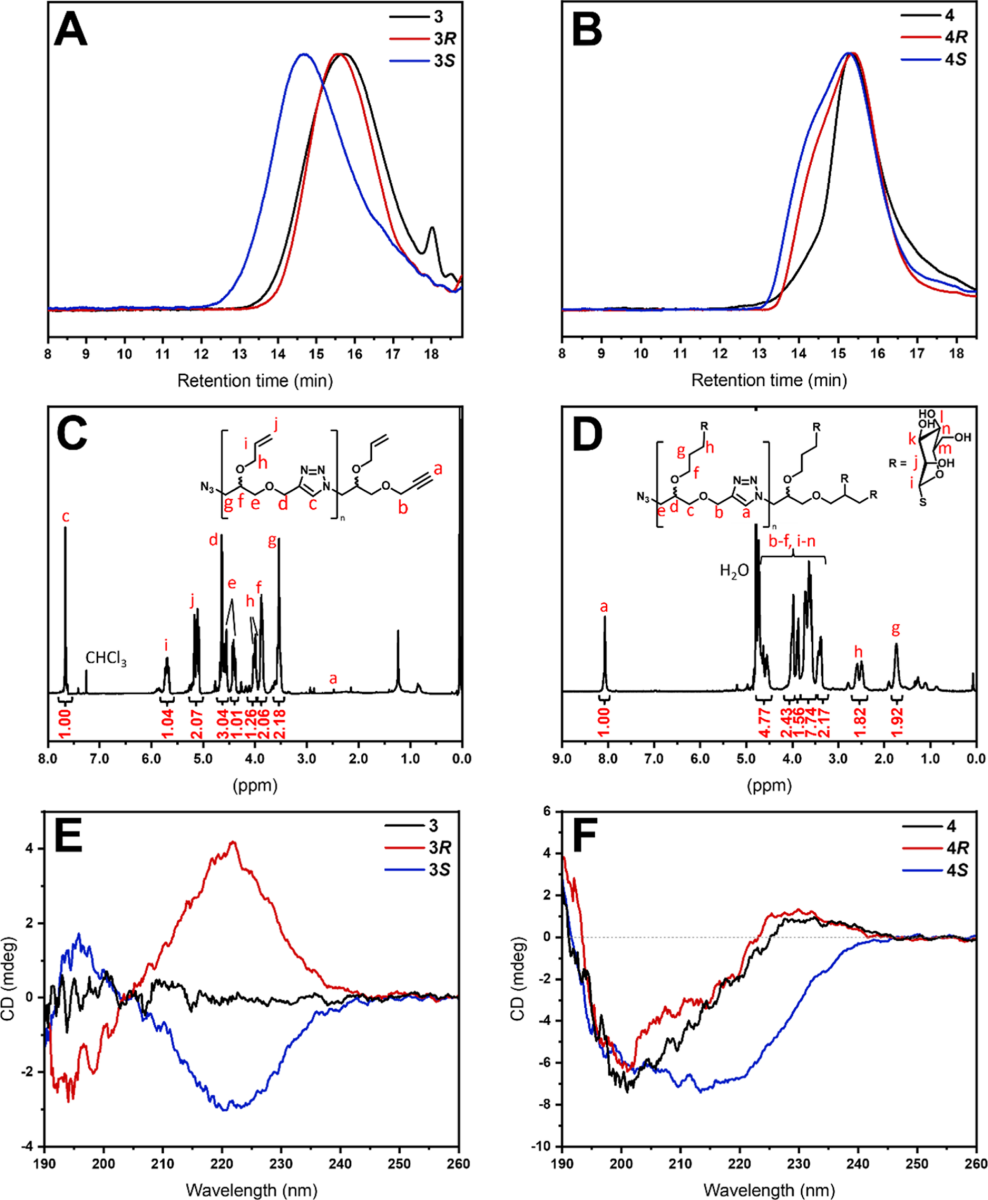
(A) GPC traces (RI detection, DMF) of allyl polytriazoles **3**, **3*R***, and **3*S***. (B) GPC traces (RI detection, DMF) of glycopolymers **4**, **4*R***, and **4*S***. (C) Representative ^1^H NMR spectrum of allyl polytriazoles (400 MHz, CDCl_3_). (D) Representative ^1^H NMR spectrum of glycopolymers (400 MHz, D_2_O). (E) Overlaid CD spectra of allyl polytriazoles **3**, **3*R***, and **3*S*** (in MeCN). (F) Overlaid CD spectra of glycopolymers **4**, **4*R***, and **4*S*** (in H_2_O).

Glycosylation of the new step-growth polytriazoles was performed through the addition of 2,3,4,6-tetra-*O*-acetyl-1-thio-β-d-mannopyranose (**Ac**_**4**_**ManSH**, see Supporting Information Figure S1) to the pendant allylic double bonds. The reaction conditions were set to a ratio of ene/thiol/AIBN = 1.0/6.0/0.25, and completion was observed by monitoring the double bond signals at 5.71 and 5.14 ppm by ^1^H NMR. Likewise, polymer **5**, which serves as a negative control for lectin binding studies, was synthesized from **3** in the same fashion. After thiol–ene addition, the acetyl groups of the crude are deacetylated in the presence of sodium methoxide. Subsequently, the final polymers are dialyzed against water to eliminate unreacted carbohydrates and then lyophilized. In the NMR spectra of the final products, no acetyl signals are detected, indicating quantitative deprotection of the carbohydrates (Figure 2). The GPC results of **4*R*** and **4*S*** suggest a similar *M*_n_ of around 12 kDa, whereas **4** shows a lower *M*_n_ of 5200 Da. The observed dispersities of the three glycopolymers range from 1.53 to 1.87. CD spectra have been acquired before and after glycosylation. No CD activity was observed for racemic polymer **3** in MeCN, whereas **3*R*** and **3*S*** rendered spectra with main bands at 222 nm. The band for **3*R*** is positive, while the spectrum of **3*S*** results in the inverted signal with a negative band, confirming the retention of stereoconfiguration throughout the polymerization process. After the thiol–ene addition of mannose units, the CD spectra change significantly, presumably due to the introduction of a chiral carbohydrate molecule to the scaffold. Racemic glycopolymer **4** appears to have a similar CD activity as **4*R***, suggesting a random coil structure since both spectra show a maximum at circa 230 nm and a minimum negative band at 201 nm. On the other hand, **4*S*** only exhibits a broad negative band with a minimum at 213 nm.

### Lectin-Binding Analysis by SPR

**Figure 3:**
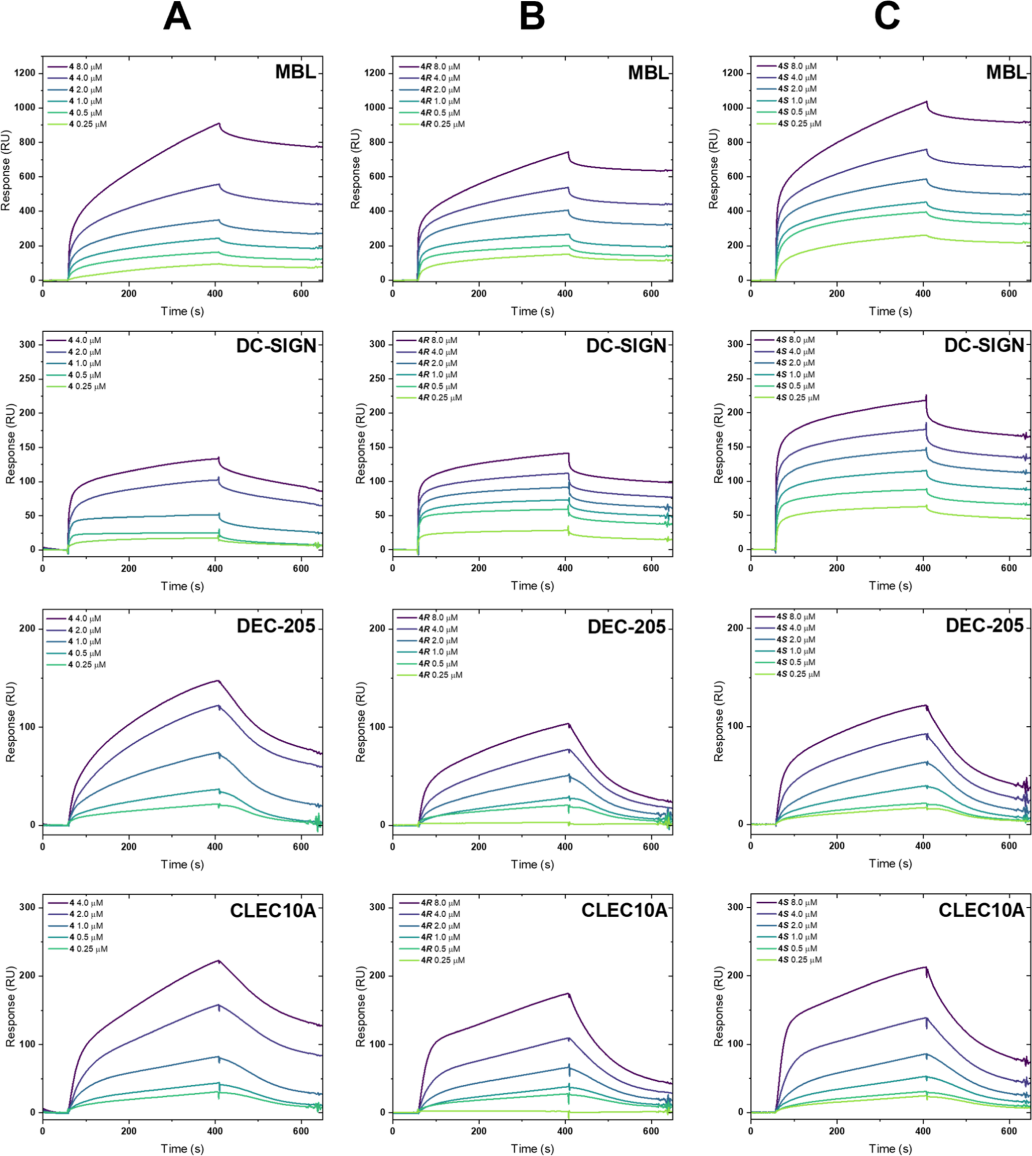
SPR binding curves of the step-growth glycopolymers (A) **4**, (B) **4*R***, and (C) **4*S*** in HBS-buffer against immobilized lectins MBL, DC-SIGN, DEC-205 (mannose-specific), and CLEC10A (galactosamine-specific). Sensorgrams for reference polymer **5** can be found in the Supporting Information.

SPR studies were conducted by immobilizing the lectins on a surface sensor chip (CM5) and subsequently flowing the glycopolymers in HBS buffer (pH = 7.4) over the substrate. Binding assays were recorded in a concentration range from 8.0 to 0.25 μM (Figure 3). It was expected to observe strong interactions between the mannose-containing polytriazoles and the three mannose-specific lectins, MBL, DC-SIGN, and DEC-205. DC-SIGN and DEC-205 are both transmembrane proteins responsible for endocytosis and antigen presentation, predominantly on dendritic cells.^38,40^ MBL belongs to a class of soluble collectins found in serum with a variety of functions in the first line of immune defense.^39,46^ In contrast, CLEC10A was chosen as a negative control due to its specificity to galactose and galactosamine. All lectins are C-type and require calcium ions to bind to carbohydrates. Furthermore, polymer **5** with pendant hydroxyl groups instead of carbohydrate moieties was prepared and screened against all lectins as a second measure to account for any nonspecific binding (see Supporting Information, Figure S34). The initial expectations were met for the binding assays of DC-SIGN and MBL, which both show significant interaction with all glycopolymers at all tested concentrations. On the contrary, only weak binding was observed for DEC-205. Especially the magnitude of nonspecific binding with **5** is much more pronounced in proportion to the glycopolymer signals. Unexpectedly, the glycopolymers also seem to exhibit binding to CLEC10A. Both the relatively high binding response of **5** and the fact that mannose is not the preferred binding partner of this receptor lead to the assumption that the measured sensorgrams for CLEC10A derive mostly from nonspecific interactions.

**Figure 4:**
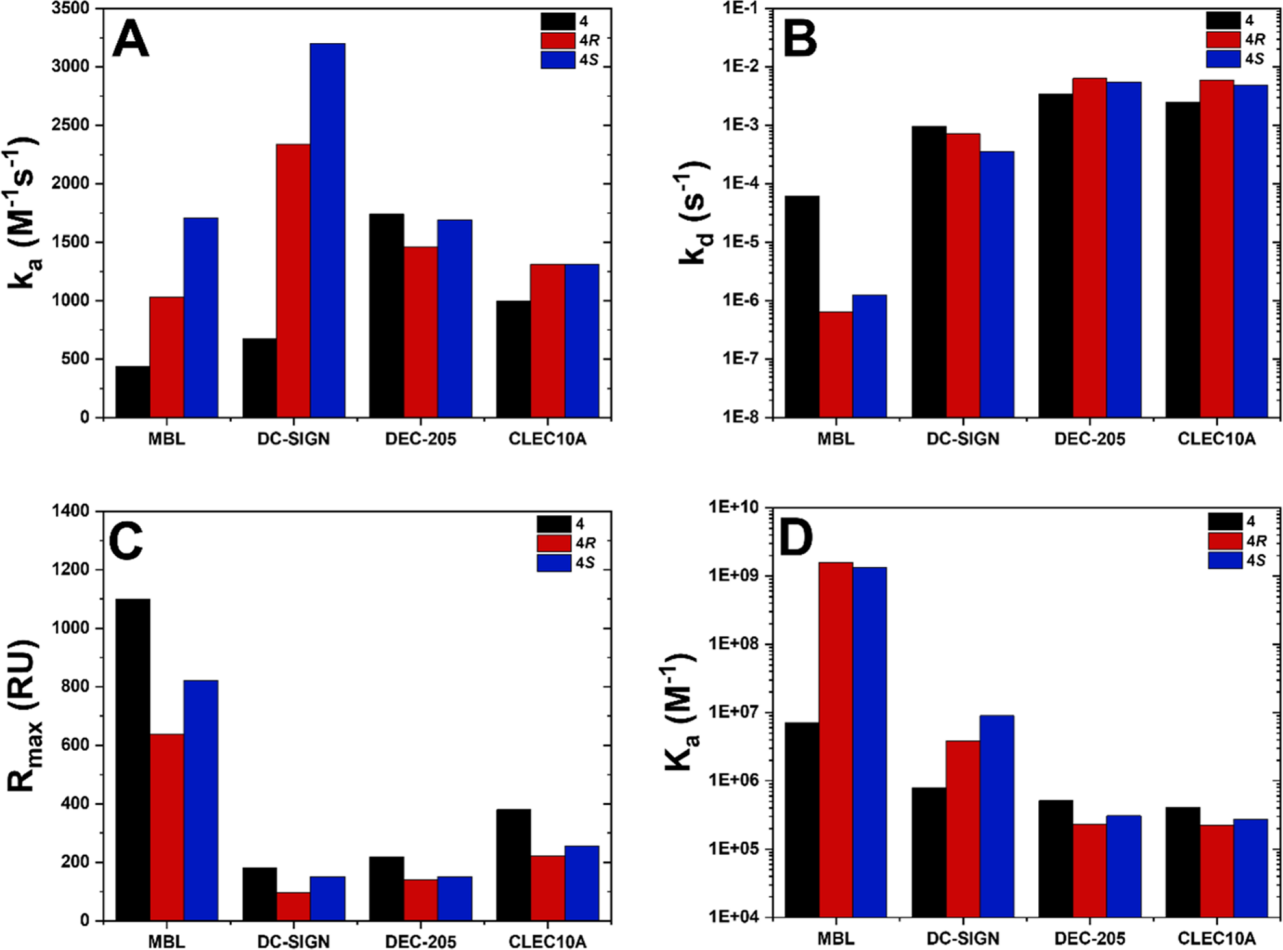
Kinetic parameters obtained from SPR binding curves by 1:1 Langmuir fitting for step-growth glycopolymers with lectins MBL, DC-SIGN, DEC-205, and CLEC10A. (A) Association rate constants (*k*_a_), (B) dissociation rate constants (*k*_d_), (C) *R*_max_ values in SPR response units, (D) association constants (*K*_a_), and (E) dissociation constants (*K*_d_).

Kinetic evaluation of the SPR results (Figure 4) reveals that polymers **4*R*** and **4*S*** bind stronger to MBL than to racemic polymer **4** by circa two orders of magnitude, with association constants (*K*_a_) of 4.50 × 10^8^ and 3.35 × 10^8^ M^–1^, respectively. Given the lower DP and hence a shorter chain length of **4*R*** compared to **4*S***, it would be expected to result in a lower affinity when binding to MBL. However, the similar association constants suggest that **4*R*** provides a preferred configuration for binding to MBL. In the case of DC-SIGN, binding constants are generally smaller than for MBL, and the magnitude of binding seems to correlate with the polymer DP (**4*S*** > **4*R*** > **4**). In contrast, association constants for DEC-205 and CLEC10A are observed in a similar magnitude among all polymers, with **4** slightly higher than the two chiral polymers. It can be assumed that both the multivalent effect and the stereoconfiguration of the backbone play only a minor role in the interaction with these lectins. In terms of association rate constants (*k*_a_), MBL and DC-SIGN show a trend of faster interaction with increasing DP of glycopolymers. The MBL disassociation rates (*k*_d_), however, appear to be significantly lower for **4*R*** and **4*S*** than for **4**. This trend is not observed for DC-SIGN, as the *k*_d_-values are within the same order of magnitude for all glycopolymers. Overall, the SPR binding experiments show that a defined stereochemistry of glycopolymers affects interactions to human lectins, yet the effect of multivalency is still prevalent as a significant characteristic for strong binding profiles.

## Conclusions

In conclusion, a set of glycopolymers derived from step-growth polymerization have been established and characterized by GPC, NMR, MALDI–ToF, and CD spectroscopy. We were able to use CuAAC of A–B-type monomers in combination with thiol–ene chemistry as an efficient tool toward a quick and facile synthesis of glycopolymers with control over the stereoconfiguration of the backbone. Binding analysis to human lectins by SPR has been conducted, revealing different affinities depending on their molecular weight as well as tacticity. From these results, it can be deduced that step-growth polymers bear a high potential to fabricate macromolecules for biomedical applications. Additionally, we are confident that control over stereoconfiguration in polymers will add to the repertoire of synthetic tools in the field to modulate selective interactions in biologic systems. Future work will be focused on the comparison between polymers and unimolecular systems, as there is still uncertainty about the role of polydispersity in multivalent binding properties.

## Supporting Information Available

Detailed experimental data, synthesis procedures, NMR spectra, MALDI–ToF data, GPC traces, and reference polymer **5**) SPR sensorgrams (PDF)

## Abbreviations

CuBr: copper(I) bromide
PMDETA: *N*,*N*,*N*′,*N*″,*N*″-pentamethyldiethylenetriamine
GPC: gel permeation chromatography
DP: degree of polymerization
CuAAC: copper-catalyzed azide–alkyne cycloaddition
SPR: surface plasmon resonance
CD: circular dichroism
MBL: mannose-binding lectin
DC-SIGN: dendritic cell-specific intercellular adhesion molecule-3-grabbing non-integrin
CLEC10A: C-type lectin 10A
DEC205: dendritic and thymic epithelial cell-205
DP: degree of polymerization
THF: tetrahydrofuran
DMF: dimethylformamide
NMR: nuclear magnetic resonance

## References

[ref1] Dwek R. A. Glycobiology: Toward Understanding the Function of Sugars. Chem. Rev. 1996, 96, 683–720. 10.1021/cr940283b. 11848770

[ref2] Bertozzi C. R.; Kiessling L. L. Chemical glycobiology. Science 2001, 291, 2357–2364. 10.1126/science.1059820. 11269316

[ref3] Hu J.; Lu K.; Gu C.; Heng X.; Shan F.; Chen G. Synthetic Sugar-Only Polymers with Double-Shoulder Task: Bioactivity and Imaging. Biomacromolecules 2022, 23, 1075–1082. 10.1021/acs.biomac.1c01409. 35089683

[ref4] Li D.; Chen J.; Hong M.; Wang Y.; Haddleton D. M.; Li G.-Z.; Zhang Q. Cationic Glycopolymers with Aggregation-Induced Emission for the Killing, Imaging, and Detection of Bacteria. Biomacromolecules 2021, 22, 2224–2232. 10.1021/acs.biomac.1c00298. 33909978

[ref5] Lis H.; Sharon N. Lectins: Carbohydrate-Specific Proteins That Mediate Cellular Recognition. Chem. Rev. 1998, 98, 637–674. 10.1021/cr940413g. 11848911

[ref6] Peacock J. S.; Colsky A. S.; Pinto V. B. Lectins and antibodies as tools for studying cellular interactions. J. Immunol. Methods 1990, 126, 147–157. 10.1016/0022-1759(90)90145-l. 2406344

[ref7] Sharon N.; Lis H. Lectins: cell-agglutinating and sugar-specific proteins. Science 1972, 177, 949–959. 10.1126/science.177.4053.949. 5055944

[ref8] Dube D. H.; Bertozzi C. R. Glycans in cancer and inflammation--potential for therapeutics and diagnostics. Nat. Rev. Drug Discovery 2005, 4, 477–488. 10.1038/nrd1751. 15931257

[ref9] Alvarez C. P.; Lasala F.; Carrillo J.; Muniz O.; Corbi A. L.; Delgado R. C-type lectins DC-SIGN and L-SIGN mediate cellular entry by Ebola virus in cis and in trans. J. Virol. 2002, 76, 6841–6844. 10.1128/jvi.76.13.6841-6844.2002. 12050398 PMC136246

[ref10] Lozach P. Y.; Amara A.; Bartosch B.; Virelizier J. L.; Arenzana-Seisdedos F.; Cosset F. L.; Altmeyer R. C-type lectins L-SIGN and DC-SIGN capture and transmit infectious hepatitis C virus pseudotype particles. J. Biol. Chem. 2004, 279, 32035–32045. 10.1074/jbc.m402296200. 15166245

[ref11] Mason C. P.; Tarr A. W. Human lectins and their roles in viral infections. Molecules 2015, 20, 2229–2271. 10.3390/molecules20022229. 25642836 PMC6272597

[ref12] Kaltner H.; Abad-Rodriguez J.; Corfield A. P.; Kopitz J.; Gabius H. J. The sugar code: letters and vocabulary, writers, editors and readers and biosignificance of functional glycan-lectin pairing. Biochem. J. 2019, 476, 2623–2655. 10.1042/bcj20170853. 31551311

[ref13] Demir Duman F.; Monaco A.; Foulkes R.; Becer C. R.; Forgan R. S. Glycopolymer-Functionalized MOF-808 Nanoparticles as a Cancer-Targeted Dual Drug Delivery System for Carboplatin and Floxuridine. ACS Appl. Nano Mater. 2022, 5, 13862–13873. 10.1021/acsanm.2c01632. 36338327 PMC9623548

[ref14] Blakney A. K.; Abdouni Y.; Yilmaz G.; Liu R.; McKay P. F.; Bouton C. R.; Shattock R. J.; Becer C. R. Mannosylated Poly(ethylene imine) Copolymers Enhance saRNA Uptake and Expression in Human Skin Explants. Biomacromolecules 2020, 21, 2482–2492. 10.1021/acs.biomac.0c00445. 32250603

[ref15] Sahkulubey Kahveci E. L.; Kahveci M. U.; Celebi A.; Avsar T.; Derman S. Glycopolymer and Poly(β-amino ester)-Based Amphiphilic Block Copolymer as a Drug Carrier. Biomacromolecules 2022, 23, 4896–4908. 10.1021/acs.biomac.2c01076. 36317475 PMC9667500

[ref16] Kiessling L. L.; Grim J. C. Glycopolymer probes of signal transduction. Chem. Soc. Rev. 2013, 42, 4476–4491. 10.1039/c3cs60097a. 23595539 PMC3808984

[ref17] Ting S. R. S.; Chen G.; Stenzel M. H. Synthesis of glycopolymers and their multivalent recognitions with lectins. Polym. Chem. 2010, 1, 1392–1412. 10.1039/c0py00141d.

[ref18] Stenzel M. H. Glycopolymers for Drug Delivery: Opportunities and Challenges. Macromolecules 2022, 55, 4867–4890. 10.1021/acs.macromol.2c00557.

[ref19] Becer C. R. The glycopolymer code: synthesis of glycopolymers and multivalent carbohydrate-lectin interactions. Macromol. Rapid Commun. 2012, 33, 742–752. 10.1002/marc.201200055. 22508520

[ref20] Abdouni Y.; ter Huurne G. M.; Yilmaz G.; Monaco A.; Redondo-Gómez C.; Meijer E. W.; Palmans A. R. A.; Becer C. R. Self-Assembled Multi- and Single-Chain Glyconanoparticles and Their Lectin Recognition. Biomacromolecules 2021, 22, 661–670. 10.1021/acs.biomac.0c01486. 33373527

[ref21] Monaco A.; Beyer V. P.; Napier R.; Becer C. R. Multi-Arm Star-Shaped Glycopolymers with Precisely Controlled Core Size and Arm Length. Biomacromolecules 2020, 21, 3736–3744. 10.1021/acs.biomac.0c00838. 32786531

[ref22] Miura Y.; Hoshino Y.; Seto H. Glycopolymer Nanobiotechnology. Chem. Rev. 2016, 116, 1673–1692. 10.1021/acs.chemrev.5b00247. 26509280

[ref23] Beyer V. P.; Monaco A.; Napier R.; Yilmaz G.; Becer C. R. Bottlebrush Glycopolymers from 2-Oxazolines and Acrylamides for Targeting Dendritic Cell-Specific Intercellular Adhesion Molecule-3-Grabbing Nonintegrin and Mannose-Binding Lectin. Biomacromolecules 2020, 21, 2298–2308. 10.1021/acs.biomac.0c00246. 32320219

[ref24] Liu L.; Zhou F.; Hu J.; Cheng X.; Zhang W.; Zhang Z.; Chen G.; Zhou N.; Zhu X. Topological Glycopolymers as Agglutinator and Inhibitor: Cyclic versus Linear. Macromol. Rapid Commun. 2019, 40, 1900223 10.1002/marc.201900223. 31241813

[ref25] Abdouni Y.; Yilmaz G.; Monaco A.; Aksakal R.; Becer C. R. Effect of Arm Number and Length of Star-Shaped Glycopolymers on Binding to Dendritic and Langerhans Cell Lectins. Biomacromolecules 2020, 21, 3756–3764. 10.1021/acs.biomac.0c00856. 32786538

[ref26] Yilmaz G.; Uzunova V.; Napier R.; Becer C. R. Single-Chain Glycopolymer Folding via Host–Guest Interactions and Its Unprecedented Effect on DC-SIGN Binding. Biomacromolecules 2018, 19, 3040–3047. 10.1021/acs.biomac.8b00600. 29870244

[ref27] Nagao M.; Kichize M.; Hoshino Y.; Miura Y. Influence of Monomer Structures for Polymeric Multivalent Ligands: Consideration of the Molecular Mobility of Glycopolymers. Biomacromolecules 2021, 22, 3119–3127. 10.1021/acs.biomac.1c00553. 34152744

[ref28] Richards S. J.; Gibson M. I. Toward Glycomaterials with Selectivity as Well as Affinity. JACS Au 2021, 1, 2089–2099. 10.1021/jacsau.1c00352. 34984416 PMC8717392

[ref29] Zhao T.; Terracciano R.; Becker J.; Monaco A.; Yilmaz G.; Becer C. R. Hierarchy of Complex Glycomacromolecules: From Controlled Topologies to Biomedical Applications. Biomacromolecules 2022, 23, 543–575. 10.1021/acs.biomac.1c01294. 34982551

[ref30] Konietzny P. B.; Freytag J.; Feldhof M. I.; Müller J. C.; Ohl D.; Stehle T.; Hartmann L. Synthesis of Homo- and Heteromultivalent Fucosylated and Sialylated Oligosaccharide Conjugates via Preactivated N-Methyloxyamine Precision Macromolecules and Their Binding to Polyomavirus Capsid Proteins. Biomacromolecules 2022, 23, 5273–5284. 10.1021/acs.biomac.2c01092. 36398945

[ref31] Hartweg M.; Jiang Y.; Yilmaz G.; Jarvis C. M.; Nguyen H. V. T.; Primo G. A.; Monaco A.; Beyer V. P.; Chen K. K.; Mohapatra S.; Axelrod S.; Gomez-Bombarelli R.; Kiessling L. L.; Becer C. R.; Johnson J. A. Synthetic Glycomacromolecules of Defined Valency, Absolute Configuration, and Topology Distinguish between Human Lectins. JACS Au 2021, 1, 1621–1630. 10.1021/jacsau.1c00255. 34723265 PMC8549053

[ref32] Baekeland L. H. Method of making insoluble products of phenol and formaldehyde. U.S. Patent 0,942,699 A, 1909.

[ref33] Besset C.; Binauld S.; Ibert M.; Fuertes P.; Pascault J.-P.; Fleury E.; Bernard J.; Drockenmuller E. Copper-Catalyzed vs Thermal Step Growth Polymerization of Starch-Derived α-Azide−ω-Alkyne Dianhydrohexitol Stereoisomers: To Click or Not To Click?. Macromolecules 2009, 43, 17–19. 10.1021/ma9024784.

[ref34] Binauld S.; Damiron D.; Hamaide T.; Pascault J. P.; Fleury E.; Drockenmuller E. Click chemistry step growth polymerization of novel alpha-azide-omega-alkyne monomers. Chem. Commun. (Cambridge, U. K.) 2008, 35, 4138–4140. 10.1039/b805164j. 18802508

[ref35] Gungor F. S.; Kiskan B. One-pot synthesis of poly(triazole-graft-caprolactone) via ring-opening polymerization combined with click chemistry as a novel strategy for graft copolymers. React. Funct. Polym. 2014, 75, 51–55. 10.1016/j.reactfunctpolym.2013.12.005.

[ref36] Besset C.; Pascault J. P.; Fleury E.; Drockenmuller E.; Bernard J. Structure-properties relationship of biosourced stereocontrolled polytriazoles from click chemistry step growth polymerization of diazide and dialkyne dianhydrohexitols. Biomacromolecules 2010, 11, 2797–2803. 10.1021/bm100872h. 20845939

[ref37] Zhang Q.; Su L.; Collins J.; Chen G.; Wallis R.; Mitchell D. A.; Haddleton D. M.; Becer C. R. Dendritic Cell Lectin-Targeting Sentinel-like Unimolecular Glycoconjugates To Release an Anti-HIV Drug. J. Am. Chem. Soc. 2014, 136, 4325–4332. 10.1021/ja4131565. 24568546

[ref38] Geijtenbeek T. B. H.; Kwon D. S.; Torensma R.; van Vliet S. J.; van Duijnhoven G. C. F.; Middel J.; Cornelissen I. L. M. H. A.; Nottet H. S. L. M.; KewalRamani V. N.; Littman D. R.; Figdor C. G.; van Kooyk Y. DC-SIGN, a Dendritic Cell–Specific HIV-1-Binding Protein that Enhances trans-Infection of T Cells. Cell 2000, 100, 587–597. 10.1016/s0092-8674(00)80694-7. 10721995

[ref39] Ji X.; Olinger G. G.; Aris S.; Chen Y.; Gewurz H.; Spear G. T. Mannose-binding lectin binds to Ebola and Marburg envelope glycoproteins, resulting in blocking of virus interaction with DC-SIGN and complement-mediated virus neutralization. J. Gen. Virol. 2005, 86, 2535–2542. 10.1099/vir.0.81199-0. 16099912

[ref40] Shrimpton R. E.; Butler M.; Morel A. S.; Eren E.; Hue S. S.; Ritter M. A. CD205 (DEC-205): a recognition receptor for apoptotic and necrotic self. Mol. Immunol. 2009, 46, 1229–1239. 10.1016/j.molimm.2008.11.016. 19135256 PMC2680960

[ref41] Geijtenbeek T. B. H.; Gringhuis S. I. Signalling through C-type lectin receptors: shaping immune responses. Nat. Rev. Immunol. 2009, 9, 465–479. 10.1038/nri2569. 19521399 PMC7097056

[ref42] Higashi N.; Fujioka K.; Denda-Nagai K.; Hashimoto S.; Nagai S.; Sato T.; Fujita Y.; Morikawa A.; Tsuiji M.; Miyata-Takeuchi M.; Sano Y.; Suzuki N.; Yamamoto K.; Matsushima K.; Irimura T. The macrophage C-type lectin specific for galactose/N-acetylgalactosamine is an endocytic receptor expressed on monocyte-derived immature dendritic cells. J. Biol. Chem. 2002, 277, 20686–20693. 10.1074/jbc.m202104200. 11919201

[ref43] Barnes J. C.; Ehrlich D. J. C.; Gao A. X.; Leibfarth F. A.; Jiang Y.; Zhou E.; Jamison T. F.; Johnson J. A. Iterative exponential growth of stereo- and sequence-controlled polymers. Nat. Chem. 2015, 7, 810–815. 10.1038/nchem.2346. 26391080

[ref44] Golder M. R.; Jiang Y.; Teichen P. E.; Nguyen H. V. T.; Wang W.; Milos N.; Freedman S. A.; Willard A. P.; Johnson J. A. Stereochemical Sequence Dictates Unimolecular Diblock Copolymer Assembly. J. Am. Chem. Soc. 2018, 140, 1596–1599. 10.1021/jacs.7b12696. 29356516 PMC5803323

[ref45] Paul C.; Hiemenz T. P. L. Polymer Chemistry, 2nd ed.; Taylor & Francis Group: Boca Raton, FL, 2007.

[ref46] Epstein J.; Eichbaum Q.; Sheriff S.; Ezekowitz R. A. B. The collectins in innate immunity. Curr. Opin. Immunol. 1996, 8, 29–35. 10.1016/s0952-7915(96)80101-4. 8729443

