# Supplementary material for: Step-Growth Glycopolymers with a Defined Tacticity for Selective Carbohydrate–Lectin Recognition
Source: Biomacromolecules. 2023 Mar 28;24(4):1924–33. doi: 10.1021/acs.biomac.3c00133 (PMC10091353; doi:10.1021/acs.biomac.3c00133)
Supplement: Supplementary file 1 — bm3c00133_si_001.pdf [file bm3c00133_si_001.pdf]

Electronic Supporting Information

Step-growth Glycopolymers with a Defined Tacticity for Selective Carbohydrate-Lectin Recognition

Jonas Becker[1], Roberto Terracciano[1], Gokhan Yilmaz[1], Richard Napier[2] and C. Remzi Becer[1]

[1]Department of Chemistry, University of Warwick, Coventry, CV4 7AL, United Kingdom

[2]School of Life Sciences, University of Warwick, Coventry CV4 7AL, United Kingdom

Remzi.Becer@warwick.ac.uk

www.becergroup.com

Table of Contents

1. Experimental Section 2

1.2 Synthesis procedures 2

1.3 Thiosugar synthesis 12

2. GPC traces of step-growth CuAAC polymerizations 13

3. Maximum DP calculation 18

4. MALDI-ToF of 3, 3*R* and 3*S* 19

5. NMR spectra 20

6. SPR data overview 30

7. SPR sensorgrams of polymer 5 31

8. IR Analysis of compounds 2, 3 and 4 32

9. References 32

1. Experimental Section

1.2 Synthesis procedures

Glycidyl propargyl ether (GPE)

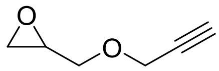

Glycidyl propargyl alcohol (GPE) was obtained from a procedure known to the literature.[1] (11.9 g, 53% yield).

(*R*)-Glycidyl propargyl ether ((*R*)-GPE)

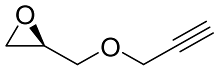

(*R*)-Glycidyl propargyl ether ((*R*)-GPE) was obtained from a procedure known to the literature.[1] (9.7 g, 53% yield).

(*S*)-Glycidyl propargyl ether ((*S*)-GPE)

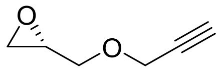

(*S*)-Glycidyl propargyl ether ((*S*)-GPE) was obtained from a procedure known to the literature.[1] (11.5 g, 63% yield).

1-azido-3-(prop-2-yn-1-yloxy)propan-2-ol (1)

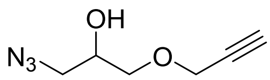

GPE (5.05 g, 45.0 mmol) was dissolved in DMF (50 mL) and acetic acid (3.86 mL, 67.5 mmol) under stirring. NaN_3 (4.39 g, 67.5 mmol) was then added in portions, and the reaction mixture was heated to 70 °C and allowed to stir for 4 h. Over the course of the reaction a white gel-like precipitate formed. After reaction completion, saturated aq. $NaHCO_3$ (300 mL) was added to neutralize any remaining acid.

It was extracted with EtOAc (3x 200 mL) and the combined organic phases were washed with saturated aq. LiCl (3x 200 mL) and then dried over $MgSO_4$. The mixture was filtered, and the solvent removed under reduced pressure. Column chromatography (hexanes/ ethyl acetate= 4:1) afforded the product **1** (2.23 g, 32% yield) as a colourless liquid.

^1H NMR (400 MHz, $CDCl_3$): δ(ppm) 4.19 (d, J = 2.4 Hz, 2H, $OC\mathit{H_2}C{\equiv}C$), 3.96 (m, 1H, $CH_2C\mathit{H}OHCH_2$), 3.57 (m, 2H, $CHC\mathit{H_2}O$), 3.38 (m, 2H, $N_3C\mathit{H_2}CH$), 2.61 (bs, 1H, $O\mathit{H}$), 2.47 (t, J = 2.4 Hz, 1H, $C{\equiv}C\mathit{H}$).

^{13}C NMR (100 MHz, $CDCl_3$): δ(ppm) 53.5 ($N_3\mathit{C}H_2$), 58.8 ($O\mathit{C}H_2C{\equiv}C$), 69.7 ($CH_2\mathit{C}HCH_2$), 71.1 ($CH\mathit{C}H_2O$), 75.3 ($\mathit{C}{\equiv}CH$), 79.4 ($C{\equiv}\mathit{C}H$).

ESI-MS for $C_6H_9N_3O_2$ $[M+Na]^+$ calc.: 178.0592, found: 178.0588.

***(R)*-1-azido-3-(prop-2-yn-1-yloxy)propan-2-ol (1*R*)**

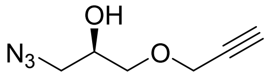

(*R*)-GPE (10.09 g, 90.0 mmol) was dissolved in DMF (100 mL) and acetic acid (8.8 mL, 135.0 mmol) under stirring. NaN_3 (7.72 g, 135.0 mmol) was then added in portions, and the reaction mixture was heated to 70 °C and allowed to stir for 4 h. Over the course of the reaction a white gel-like precipitate formed. After reaction completion, saturated aq. $NaHCO_3$ (300 mL) was added to neutralize any remaining acid. It was extracted with EtOAc (3x 200 mL) and the combined organic phases were washed with 1 M aq. LiCl (3x 200 mL) and then dried over $MgSO_4$. It was filtered and the solvent was removed under reduced pressure. Column chromatography (hexanes/ ethyl acetate= 4:1) afforded the product **1*R*** (10.4 g, 75% yield) as a colourless liquid.

^1H NMR (400 MHz, $CDCl_3$): δ(ppm) 4.18 (d, J = 2.4 Hz, 2H, $OC\mathit{H_2}C{\equiv}C$), 3.96 (m, 1H, $CH_2C\mathit{H}OHCH_2$), 3.57 (m, 2H, $CHC\mathit{H_2}O$), 3.37 (m, 2H, $N_3C\mathit{H_2}CH$), 3.07 (bs, 1H, $O\mathit{H}$), 2.47 (t, J = 2.4 Hz, 1H, $C{\equiv}C\mathit{H}$).

^{13}C NMR (100 MHz, $CDCl_3$): δ(ppm) 53.5 (N_3*C*H_2), 58.7 (O*C*H_2C≡C), 69.6 (CH_2*C*HCH_2), 71.1 (CH*C*H_2O), 75.2 (*C*≡CH), 79.3 (C≡*C*H).

ESI-MS for $C_6H_9N_3O_2$ $[M+Na]^+$ calc.: 178.0592, found: 178.0587.

***(S)*-1-azido-3-(prop-2-yn-1-yloxy)propan-2-ol (1*S*)**

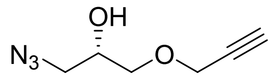

(*S*)-GPE (10.09 g, 90.0 mmol) was dissolved in DMF (100 mL) and acetic acid (8.8 mL, 135.0 mmol) under stirring. NaN_3 (7.72 g, 135.0 mmol) was then added in portions, and the reaction mixture was heated to 70 °C and allowed to stir for 4 h. Over the course of the reaction a white gel-like precipitate formed. After reaction completion, saturated aq. $NaHCO_3$ (300 mL) was added to neutralize any remaining acid. It was extracted with EtOAc (2x 300 mL) and the combined organic phases were washed with 1 M aq. LiCl (3x 200 mL) and then dried over $MgSO_4$. It was filtered and the solvent was removed under reduced pressure. Column chromatography (hexanes/ ethyl acetate= 4:1) afforded the product **1*S*** (9.5 g, 68% yield) as a colourless liquid.

^1H NMR (400 MHz, $CDCl_3$): δ(ppm) 4.19 (d, $J = 2.4$ Hz, 2H, O*C*H_2C≡C), 3.96 (m, 1H, CH_2C*H*OHCH_2), 3.57 (m, 2H, CHCH_2O), 3.38 (m, 2H, N_3CH_2CH), 2.61 (bs, 1H, O*H*), 2.47 (t, $J = 2.4$ Hz, 1H, C≡C*H*).

^{13}C NMR (100 MHz, $CDCl_3$): δ(ppm) 53.5 (N_3*C*H_2), 58.7 (O*C*H_2C≡C), 69.6 (CH_2*C*HCH_2), 71.1(CH*C*H_2O), 75.2 (*C*≡CH), 79.3 (C≡*C*H).

ESI-MS for $C_6H_9N_3O_2$ $[M+Na]^+$ calc.: 178.0592, found: 178.0586.

3-((1-azido-3-(prop-2-yn-1-yloxy)propan-2-yl)oxy)prop-1-ene (2)

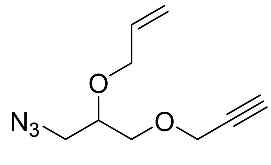

Under an N_2 atmosphere, dry DMF (25 mL) and allyl bromide (3.25 mL, 37.5 mmol) were added to **1** (3.88 g, 25.0 mmol) in an oven-dried 100 mL Schlenk-RBF. The reaction mixture was cooled to 0 °C and NaH (1.10 g, 27.5 mmol, 60% dispersion in mineral oil) was added portion-wise into the stirring reaction mixture. The mixture was allowed to gradually warm up to room temperature and left to react overnight. After completion, DMF was removed under reduced pressure. 300 mL of water was added to the solution which was extracted with DCM (3x 300 mL). The combined organic layers were washed with 1 M aq. LiCl (3x 300 mL), dried over $MgSO_4$, filtered, and the solvent removed under reduced pressure. Column chromatography (hexanes/ ethyl acetate= 20:1) afforded the product **2** (3.9 g, 80% yield) as a colourless liquid.

^{1}H NMR (400 MHz, $CDCl_3$): δ(ppm) 5.93 (ddt, *J* = 16.4, 10.9, 5.7 Hz, 1H, C*H*=CH_2), 5.31 (d, *J* = 17.2, 1H, CH=CH_2), 5.20 (d, *J* = 10.4, 1H, CH=CH_2), 4.18 (d, *J* = 2.4 Hz, 2H, OCH_2C≡C), 4.14 (m, 2H, OCH_2C=C), 3.68 (m, 1H, CH_2C*H*O), 3.61 (m, 2H, CHCH_2O), 3.38 (d, *J* = 5.0 Hz, 2H, N_3CH_2), 2.45 (t, *J* = 2.4 Hz, 1H, C≡C*H*).

^{13}C NMR (100 MHz, $CDCl_3$): δ(ppm) 52.0 (N_3*C*H_2), 58.8 (O*C*H_2C≡C), 69.2 (O*C*H_2C=C), 71.5 (CH*C*H_2O), 75.0 (*C*≡CH), 76.9 (C≡*C*H), 79.4 (CH_2*C*HCH_2), 117.6 (CH=*C*H_2), 134.5 (*C*H=CH_2).

ESI-MS for $C_9H_{13}N_3O_2$ $[M+Na]^+$ calc.: 218.0905, found: 218.0899.

***(R)*-3-((1-azido-3-(prop-2-yn-1-yloxy)propan-2-yl)oxy)prop-1-ene (2*R*)**

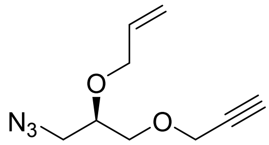

Under an N_2 atmosphere, dry DMF (25 mL) and allyl bromide (4.22 mL, 48.8 mmol) were added to **1*R*** (5.04 g, 32.5 mmol) in an oven-dried 100 mL Schlenk-RBF. The reaction mixture was cooled to 0 °C and NaH (1.43 g, 35.8 mmol, 60% dispersion in mineral oil) was added portion-wise into the stirring reaction mixture. The mixture was allowed to gradually warm up to room temperature and left to react overnight. After completion, DMF was removed under reduced pressure. 300 mL of water was added to the solution which was extracted with DCM (3x 300 mL). The combined organic layers were

washed with 1 M aq. LiCl (3x 300 mL), dried over $MgSO_4$, filtered, and the solvent removed under reduced pressure. Column chromatography (hexanes/ ethyl acetate= 20:1) afforded the product ***2R*** (2.87 g, 45% yield) as a colourless liquid.

^1H NMR (400 MHz, $CDCl_3$): δ(ppm) 5.93 (m, 1H, C*H*=CH_2), 5.31 (d, *J* = 17.2 Hz, 1H, CH=CH_2), 5.20 (d, *J* = 10.4 Hz, 1H, CH=CH_2), 4.18 (d, *J* = 1.8 Hz, 2H, OCH_2C≡C), 4.14 (m, 2H, OCH_2C=C), 3.68 (m, 1H, CH_2C*H*O), 3.61 (m, 2H, CHCH_2O), 3.38 (d, *J* = 5.0 Hz, 2H, N_3CH_2), 2.45 (t, *J* = 2.0 Hz, 1H, C≡C*H*).

^{13}C NMR (100 MHz, $CDCl_3$): δ(ppm) 52.0 (N_3*C*H_2), 58.8 (O*C*H_2C≡C), 69.2 (O*C*H_2C=C), 71.5 (CH*C*H_2O), 75.0 (*C*≡CH), 76.9 (C≡*C*H), 79.4 (CH_2*C*HCH_2), 117.6 (CH=*C*H_2), 134.6 (*C*H=CH_2).

ESI-MS for $C_9H_{13}N_3O_2$ $[M+Na]^+$ calc.: 218.0905, found: 218.0899.

***(S)*-3-((1-azido-3-(prop-2-yn-1-yloxy)propan-2-yl)oxy)prop-1-ene (2*S*)**

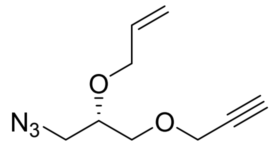

Under an N_2 atmosphere, dry DMF (25 mL) and allyl bromide (4.22 mL, 48.8 mmol) were added to ***1S*** (5.04 g, 32.5 mmol) in an oven-dried 100 mL Schlenk-RBF. The reaction mixture was cooled to 0 °C and NaH (1.43 g, 35.8 mmol, 60% dispersion in mineral oil) was added portion-wise into the stirring reaction mixture. The mixture was allowed to gradually warm up to room temperature and left to react overnight. After completion, DMF was removed under reduced pressure. 300 mL of water was added to the solution was extracted with DCM (3x 300 mL). The combined organic layers were washed with 1 M aq. LiCl (3x 300 mL), dried over $MgSO_4$, filtered, and the solvent removed under reduced pressure. Column chromatography (hexanes/ ethyl acetate= 20:1) afforded the product ***2S*** (2.60 g, 41% yield) as a colourless liquid.

^1H NMR (400 MHz, $CDCl_3$): δ(ppm) 5.93 (m, 1H, C*H*=CH_2), 5.30 (dd, *J* = 17.2, 3.1 Hz, 1.5 Hz, 1H, CH=CH_2), 5.20 (dd, *J* = 10.4, 1.4 Hz, 1H, CH=CH_2), 4.17 (d, *J* = 2.0 Hz, 2H, OCH_2C≡C), 4.13 (m, 2H,

OCH_2C=C), 3.67 (m, 1H, CH_2CHO), 3.61 (m, 2H, $CHCH_2O$), 3.37 (d, J = 5.0 Hz, 2H, N_3CH_2), 2.45 (t, J = 2.2 Hz, 1H, C≡C*H*).

^{13}C NMR (100 MHz, $CDCl_3$): δ(ppm) 52.0 (N_3CH_2), 58.8 (OCH_2C≡C), 69.2 (OCH_2C=C), 71.5 ($CHCH_2O$), 75.0 (*C*≡CH), 76.9 (C≡*C*H), 79.4 (CH_2CHCH_2), 117.6 (CH=CH_2), 134.5 (*C*H=CH_2).

ESI-MS for $C_9H_{13}N_3O_2$ $[M+Na]^+$ calc.: 218.0905, found: 218.0898.

Polymerisation of 2

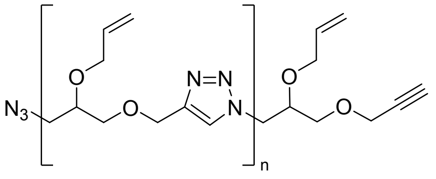

Under an N_2-atmosphere, **2** (0.50 g, 2.55 mmol) and PMDETA (0.05 mL, 0.25 mmol) were dissolved in anhydrous THF or DMF (2.2 mL) in an oven-dried Schlenk tube. The mixture was heated to 45 °C and degassed for 15 min. Then, CuBr (0.019 g, 0.13 mmol) was added, and the mixture was stirred for 4h. The mixture was precipitated into cold diethyl ether (200 mL) and filtered. The residue was dissolved in DCM (20 mL) and washed with H_2O (3x 20 mL). The organic phase was dried over $MgSO_4$, filtered, and the solvent removed under reduced pressure. Polymer **3** was obtained as an amber solid (0.302 g, 60%).

^{1}H NMR (400 MHz, $CDCl_3$): δ(ppm) 7.68 (s, 1H, H^{Ar}), 5.71 (m, 1H, C*H*=CH_2), 5.14 (m, 2H, CH=CH_2), 4.66 (s, 2H, OCH_2Ar), 4.58 (m, 1H, $CHCH_2O$), 4.43 (m, 1H, $CHCH_2O$), 4.03 (m, 1H, OCH_2C=C), 3.89 (m, 2H, OCH_2C=C + CH_2CHOCH_2), 3.54 (m, 2H, NCH_2CH).

Polymerisation of 2*R*

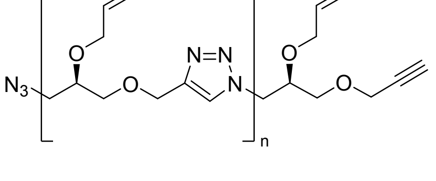

Under an N_2-atmosphere, **M1** (0.113 g, 0.58 mmol) and PMDETA (0.01 mL, 0.06 mmol) were dissolved in anhydrous DMF (0.5 mL) in an oven-dried Schlenk tube. The mixture was heated to 45 °C

and degassed for 15 min. Then, CuBr (0.004 g, 0.03 mmol) was added, and the mixture was stirred for up to 18h. The mixture was precipitated into cold diethyl ether (200 mL) and filtered. The residue was dissolved in DCM (20 mL) and washed with H_2O (3x 20 mL). The organic phase was dried over $MgSO_4$, filtered, and the solvent removed under reduced pressure. Polymer **3*R*** was obtained as an amber solid (0.047 g, 42%).

^{1}H NMR (400 MHz, $CDCl_3$): δ(ppm) 7.66 (s, 1H, H^{Ar}), 5.70 (m, 1H, C*H*=CH_2), 5.15 (d, *J* = 17.1 Hz, 1H, CH=CH_2), 5.09 (d, *J* = 10.4 Hz, 1H, CH=CH_2), 4.70-4.51 (m, 3H, OCH_2Ar + CHCH_2O), 4.40 (dd, *J* = 14.0, 7.4 Hz, 1H, CHCH_2O), 4.01 (dd, *J* = 12.7, 5.4 Hz, 1H, OCH_2C=C), 3.87 (m, 2H, OCH_2C=C + CH_2C*H*OCH_2), 3.54 (m, 2H, NCH_2CH).

Polymerisation of 2*S*

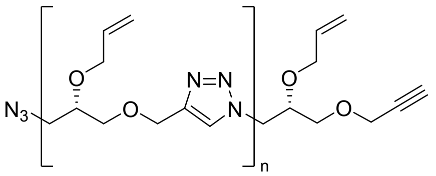

Under an N_2-atmosphere, **M1** (0.113 g, 0.58 mmol) and PMDETA (0.01 mL, 0.06 mmol) were dissolved in anhydrous DMF (0.5 mL) in an oven-dried Schlenk tube. The mixture was heated to 45 °C and degassed for 15 min. Then, CuBr (0.004 g) was added, and the mixture was stirred for up to 18h. The mixture was precipitated into cold diethyl ether (200 mL) and filtered. The residue was dissolved in DCM (20 mL) and washed with H_2O (3x 20 mL). The organic phase was dried over $MgSO_4$, filtered, and the solvent removed under reduced pressure. Polymer **3*S*** was obtained as an amber solid (0.047 g, 42%).

^{1}H NMR (400 MHz, $CDCl_3$): δ(ppm) 7.66 (s, 1H, H^{Ar}), 5.70 (m, 1H, C*H*=CH_2), 5.15 (d, *J* = 17.3 Hz, 1H, CH=CH_2), 5.09 (d, *J* = 10.3 Hz, 1H, CH=CH_2), 4.70-4.51 (m, 3H, OCH_2Ar + CHCH_2O), 4.40 (dd, *J* = 14.0, 7.2 Hz, 1H, CHCH_2O), 4.01 (dd, *J* = 12.5, 5.1 Hz, 1H, OCH_2C=C), 3.87 (m, 2H, OCH_2C=C + CH_2C*H*OCH_2), 3.53 (m, 2H, NCH_2CH).

Thiol-ene glycosylation of 3

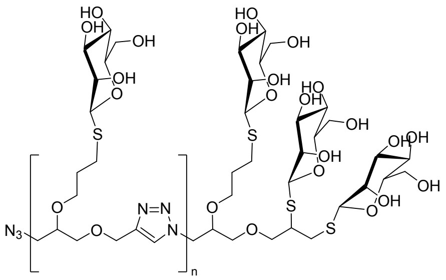

Under an N_2 atmosphere, **3** (0.092 g) was dissolved in anhydrous acetonitrile (1 mL). Ac_4Man-SH (1.03 g, 2.82 mmol, 6.00 eq per ene) and AIBN (0.02 g, 0.12 mmol, 0.25 eq per ene) were added, and the suspension was degassed for 15 min. The mixture was stirred at 70 °C for 18h and then poured into cold diethyl ether (200 mL) and filtered. The residue was collected and dried under reduced pressure. The obtained crude intermediate product was dissolved in MeOH (10 mL) and sodium methoxide (0.04 g, 0.71 mmol, 1.5 eq per mannose unit) was added and the mixture was stirred at ambient temperature. After 18h, the solvent was removed under reduced pressure and the crude product was dissolved in H_2O (6 mL). The solution was transferred into a dialysis bag (MWCO: 1 kDa) and dialyzed against H_2O for 2 days. Lyophilization afforded the product **4** (0.041 g, 22%) as a white solid.

^{1}H NMR (400 MHz, D_2O): δ(ppm) 8.08 (s, 1H, H^{Ar}), 5.01-4.44 (m, 3H, OCH_2CAr+H^1+CHCH_2O), 4.13-3.94 (m, 2H, OCHCH_2CH$_2$), 3.93-3.81 (m, 2H, CH$_2$CHOCH$_2$+H^2), 3.77-3.50 (m, 6H, H^3+H^4+H^5+H^6), 3.49-3.24 (m, 2H, NCH_2CH), 2.67-2.34 (m, 2H, SCH_2CH$_2$), 1.84-1.58 (m, 2H, CH$_2$CH_2CH$_2$).

Thiol-ene glycosylation of 3*R*

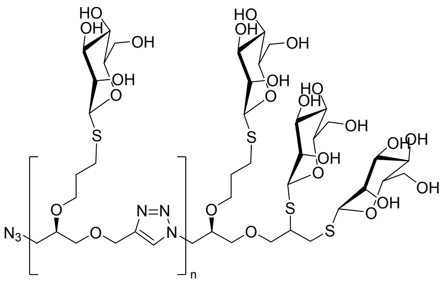

Under an N_2 atmosphere, **3*R*** (0.039 g) was dissolved in anhydrous acetonitrile (1 mL). Ac_4Man-SH (0.437 g, 1.20 mmol, 6.00 eq per ene) and AIBN (0.008 g, 0.05 mmol, 0.25 eq per ene) were added, and the suspension was degassed for 15 min. The mixture was stirred at 70 °C for 18h and then poured

into cold diethyl ether (200 mL) and filtered. The residue was collected and dried under reduced pressure. The obtained crude intermediate product was dissolved in MeOH (10 mL) and sodium methoxide (0.016 g, 0.30 mmol, 1.5 eq per mannose unit) was added and the mixture was stirred at ambient temperature. After 18h, the solvent was removed under reduced pressure and the crude product was dissolved in H_2O (6 mL). The solution was transferred into a dialysis bag (MWCO: 1 kDa) and dialyzed against H_2O for 2 days. Lyophilization afforded the product **4*R*** (0.049 g, 63%) as a white solid.

^{1}H NMR (400 MHz, D_2O): δ(ppm) 8.07 (s, 1H, H^{Ar}), 4.84-4.48 (m, 5H, $OCH_2CAr+H^1+CHCH_2O$), 4.10-3.94 (m, 2H, $OCHCH_2CH_2$), 3.93-3.82 (m, 2H, $CH_2CHOCH_2+H^2$), 3.79-3.51 (m, 6H, $H^3+H^4+H^5+H^6$), 3.50-3.31 (m, 2H, NCH_2CH), 2.66-2.42 (m, 2H, SCH_2CH_2), 1.83-1.61 (m, 2H, $CH_2CH_2CH_2$).

Thiol-ene glycosylation of 3*S*

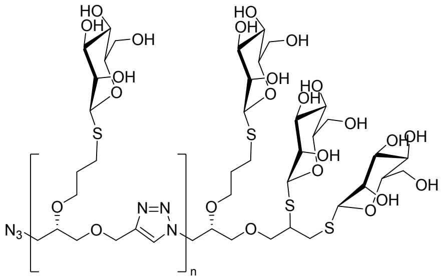

Under an N_2 atmosphere, **3*S*** (0.035 g) was dissolved in anhydrous acetonitrile (1 mL). Ac_4Man-SH (0.394 g, 1.08 mmol, 6.00 eq per ene) and AIBN (0.007 g, 0.045 mmol, 0.25 eq per ene) were added, and the suspension was degassed for 15 min. The mixture was stirred at 70 °C for 18h and then poured into cold diethyl ether (200 mL) and filtered. The residue was collected and dried under reduced pressure. The obtained crude intermediate product was dissolved in MeOH (10 mL) and sodium methoxide (0.015 g, 0.27 mmol, 1.5 eq per mannose unit) was added and the mixture was stirred at ambient temperature. After 18h, the solvent was removed under reduced pressure and the crude product was dissolved in H_2O (6 mL). The solution was transferred into a dialysis bag (MWCO: 1 kDa) and dialysed against H_2O for 2 days. Lyophilization afforded the product **4*S*** (0.065 g, 92%) as a white solid.

^{1}H NMR (400 MHz, D_2O): δ(ppm) 8.08 (s, 1H, H^{Ar}), 4.89-4.46 (m, 4H, OCH_2CAr+H^1+CHCH_2O), 4.12-3.95 (m, 2H, OCHCH_2CH$_2$), 3.94-3.82 (m, 2H, CH$_2$CHOCH$_2$+H^2), 3.79-3.51 (m, 6H, H^3+H^4+H^5+H^6), 3.50-3.31 (m, 2H, NCH_2CH), 2.65-2.43 (m, 2H, SCH_2CH$_2$), 1.83-1.61 (m, 2H, CH$_2$CH_2CH$_2$).

Reference polymer 5

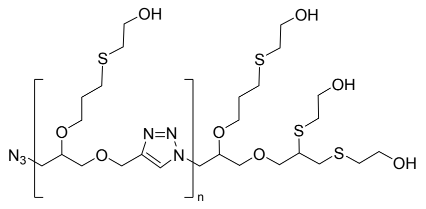

Under an N_2 atmosphere, **3** (0.053 g) was dissolved in anhydrous acetonitrile (1 mL). 2-Mercaptoethanol (0.114 mL, 1.62 mmol, 6.00 eq per ene) and AIBN (0.011 g, 0.07 mmol, 0.25 eq per ene) were added, and the suspension was degassed for 15 min and stirred at 70 °C for 18h. The mixture was allowed to cool to r.t. and a mixture of H_2O/DMSO = 1/1 (v/v) (5 mL) was added. The solution was transferred into a dialysis bag (MWCO: 1 kDa) and dialysed against H_2O for 2 days. Lyophilization afforded the product **5** (0.015 g, 20%) as a white solid.

^{1}H NMR (400 MHz, DMSO-*d6*): δ(ppm) 8.04 (s, 1H, H^{Ar}), 4.74 (t, J = 5.1 Hz, 1H), 4.63-4.47 (m, 3H, OCH_2CAr+CHCH_2O^A), 4.38 (dd, 1H, CHCH_2O^B), 3.83 (s, 1H, CH$_2$CHOCH$_2$), 3.63-3.40 (m, 6H, OCHCH_2CH$_2$+CH$_2$CH_2OH+NCH_2CH), 2.58-2.44 (m, 2H, SCH_2CH$_2$), 2.35 (dd, J = 13.1, 5.1 Hz, 2H, SCH_2CH$_2$), 1.64-1.49 (m, 2H, CH$_2$CH_2CH$_2$).

1.3 Thiosugar synthesis

2,3,4,6-Tetra-*O*-acetyl-1-thio-β-D-mannopyranose (**Ac_4ManSH**) was synthesized from procedures known to the literature as shown in **Figure S1**.[2, 3]

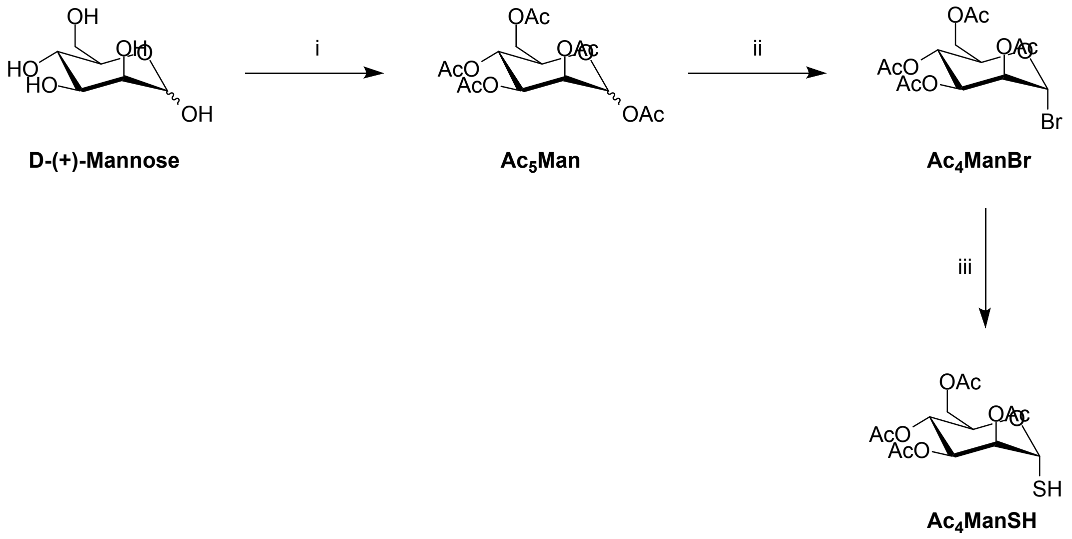

Figure S1: Synthesis scheme for the preparation of **Ac_4ManSH**, i) Ac_2O, H_2SO_4 (cat.), DCM, 0 °C to r.t., 18 h ii) HBr (33%) in AcOH, DCM, r.t., 18 h iii) $Na_2S\cdot 9H_2O$, CS_2, DMF, r.t. 15 min.

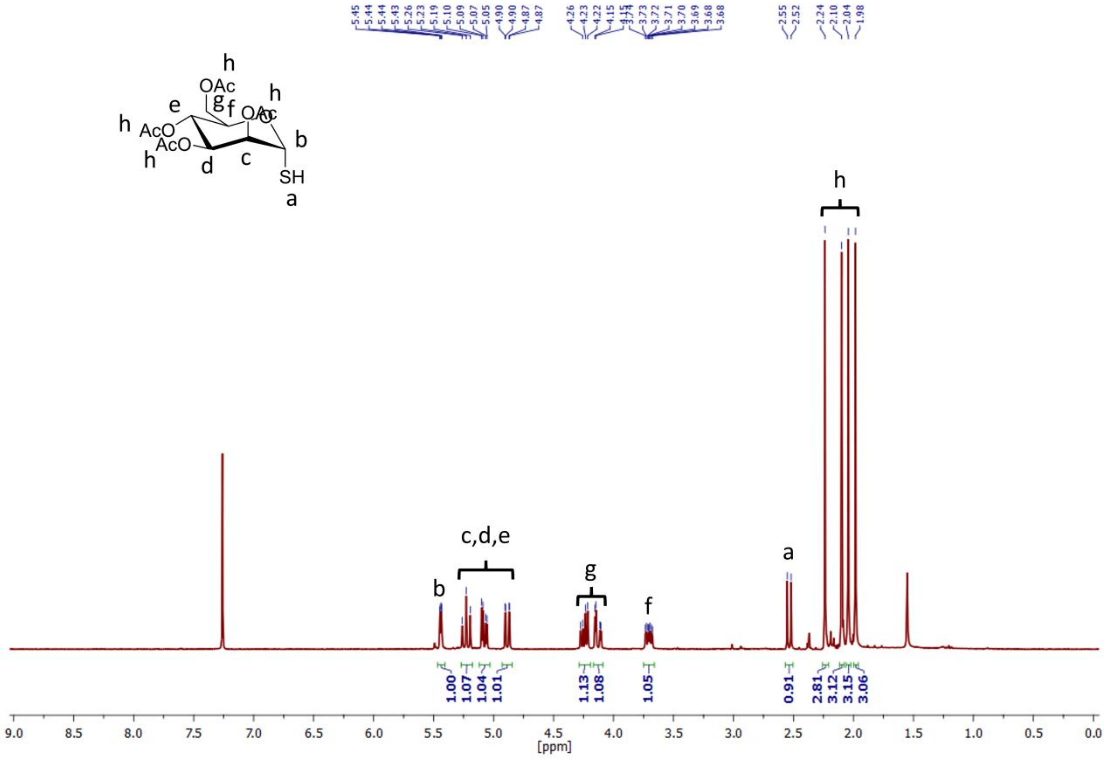

Figure S2: ^{1}H NMR spectrum (400 MHz, $CDCl_3$) of 2,3,4,6-Tetra-*O*-acetyl-1-thio-β-D-mannopyranose.

2. GPC traces of step-growth CuAAC polymerizations

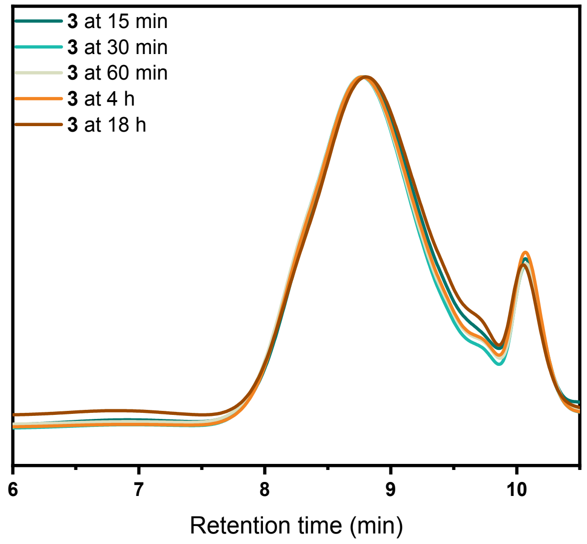

Figure S3: GPC-traces (RI-detection, THF) of **3** after polymerization in THF, 45 °C, CuBr (5 mol%), PMDETA (10 mol%), 4 h, c = 225 mg/mL over 15 min to 18 h.

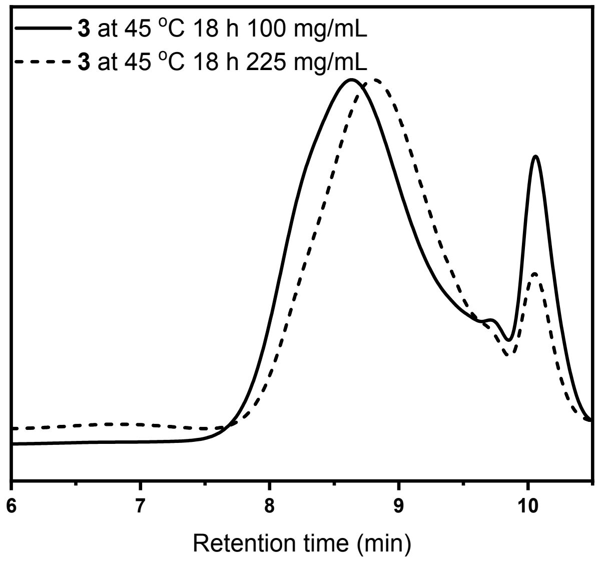

Figure S4:GPC-traces (RI-detection, THF) of **3** after polymerization at c = 100 mg/mL (solid) or c = 225 mg/mL (dashed), remaining conditions: THF, 45 °C, CuBr (5 mol%), PMDETA (10 mol%), 18 h.

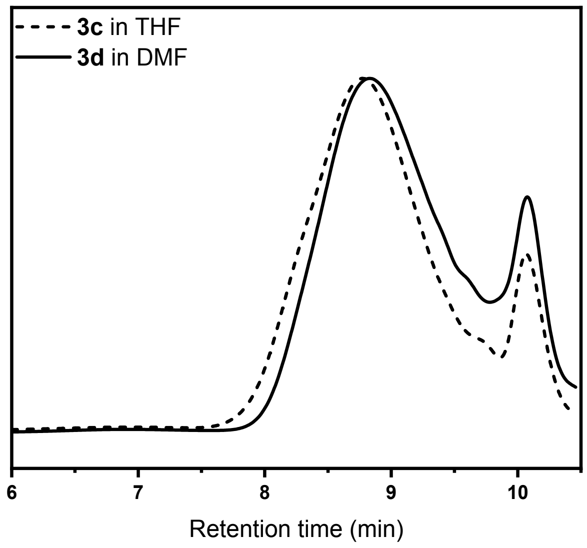

Figure S5: GPC-traces (RI-detection, THF) of **3** after polymerization in DMF (solid) or THF (dashed), remaining conditions: 45 °C, CuBr (5 mol%), PMDETA (10 mol%), 4 h, c = 225 mg/mL.

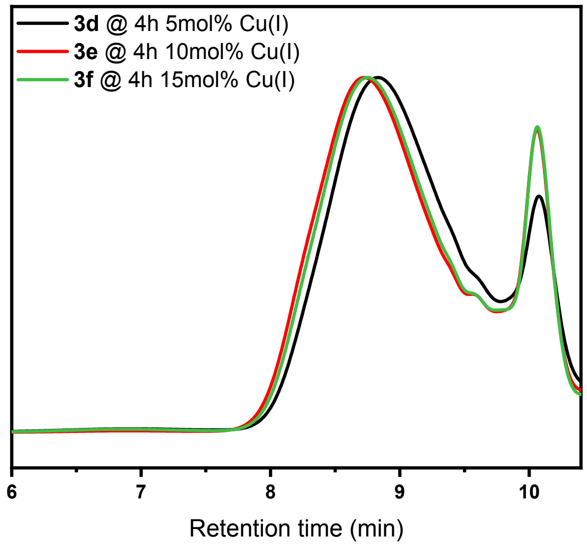

Figure S6: GPC-traces (RI-detection, THF) of **3** after polymerization in DMF, 45 °C, 4 h, c = 225 mg/mL at varying catalyst concentrations.

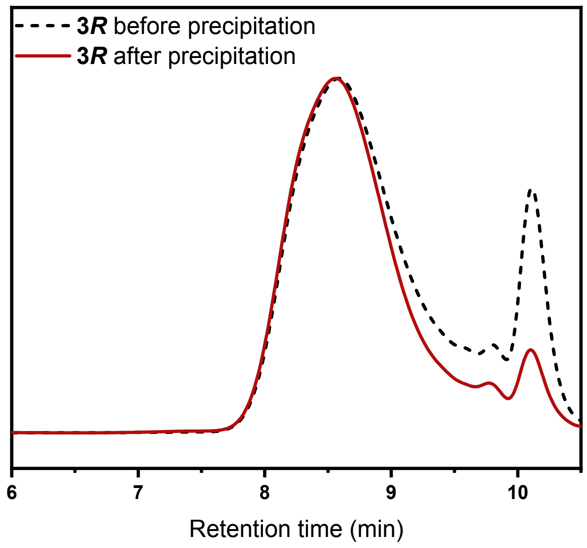

Figure S7: GPC-traces (RI-detection, THF) of **3*R*** before (dashed) and after (solid) purification by precipitation into diethyl ether.

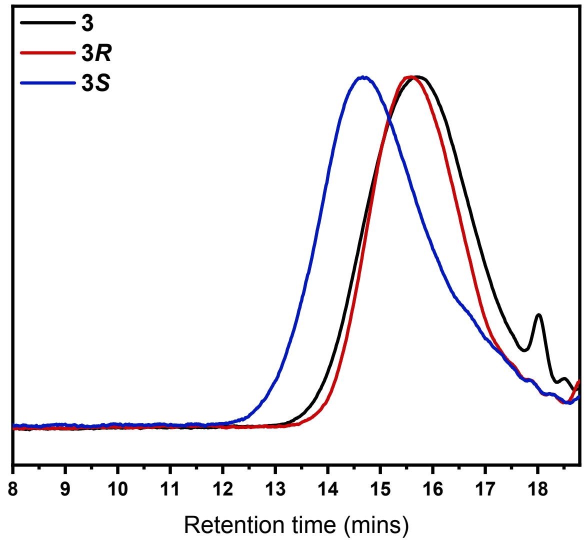

Figure S8: GPC traces (RI-detection, DMF) of polymers **3**, **3*R***, and **3*S*** after purification.

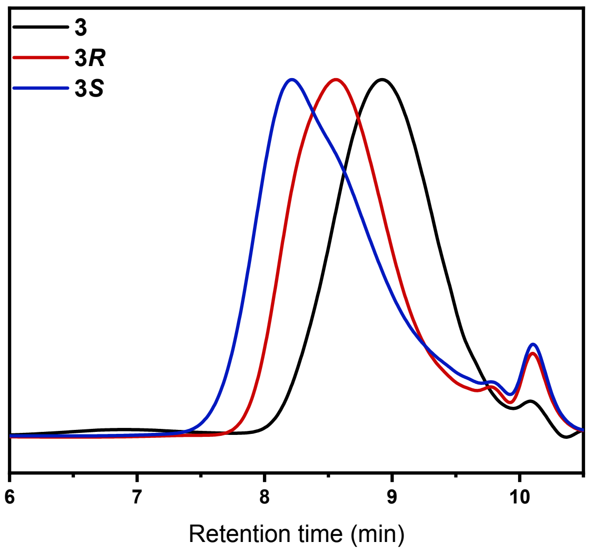

Figure S9: GPC traces (RI-detection, THF) of polymers **3**, **3*R***, and **3*S*** after purification.

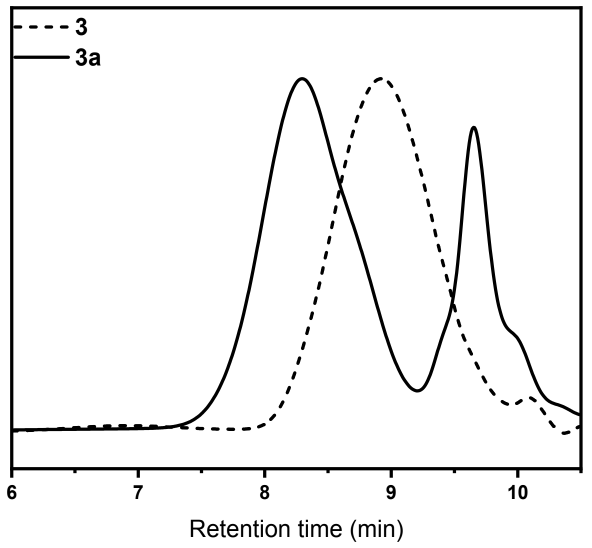

Figure S10: GPC traces (RI-detection, THF) of polymers **3** (dashed) and **3a** (solid) after thiol-ene glycosylation.

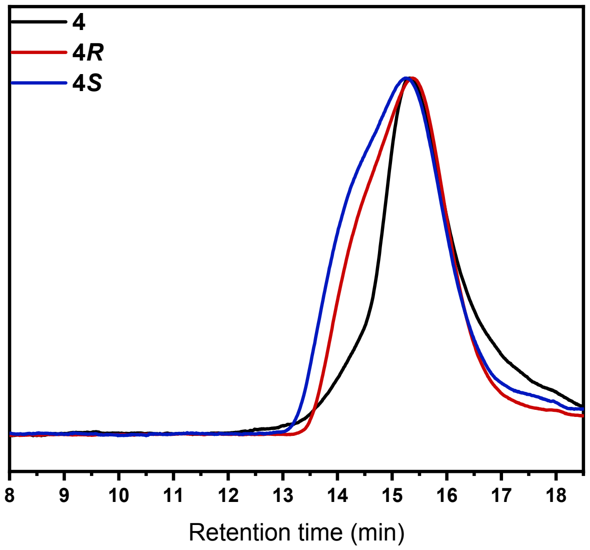

Figure S11: GPC traces (RI-detection, DMF) of glycopolymers **4**, **4*R***, and **4*S*** after deprotection and purification.

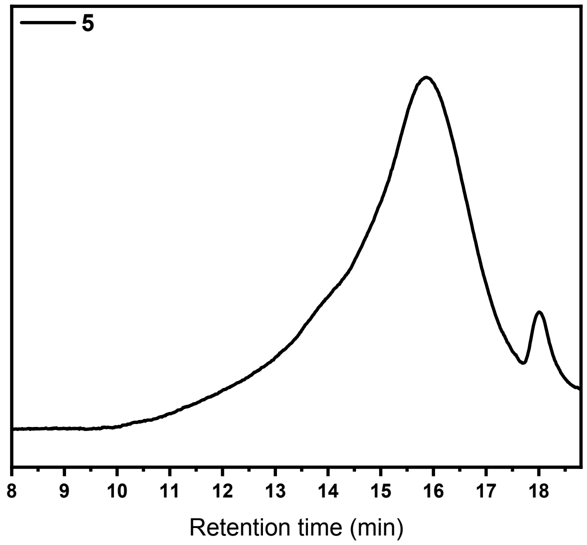

Figure S12: GPC trace (RI-detection, DMF) of reference polymer **5** after purification.

3. Maximum DP calculation

Carother's equation:

$$\overline{DP_n} = \frac{1+r}{1+r-2pr} \qquad \textbf{(I)}$$

$$\overline{DP_n} = \frac{1+r}{1-r} \qquad \textbf{(II)}$$

$$r = \frac{N_A}{N_B + N_{B'}} \qquad \textbf{(III)}$$

Table S1. Calculated theoretical maximum average DP values for step-growth polymerization of monomers **2**, **2*R*** and **2*S*** using equation (**II**), stochiometric imbalance was derived from ^{1}H NMR integrals.

Monomer	calc. *r*	calc. avg. $DP_{n, max}$
**2**	0.905	20
**2*R***	0.975	79
**2*S***	0.966	58

4. MALDI-ToF of 3, 3*R* and 3*S*

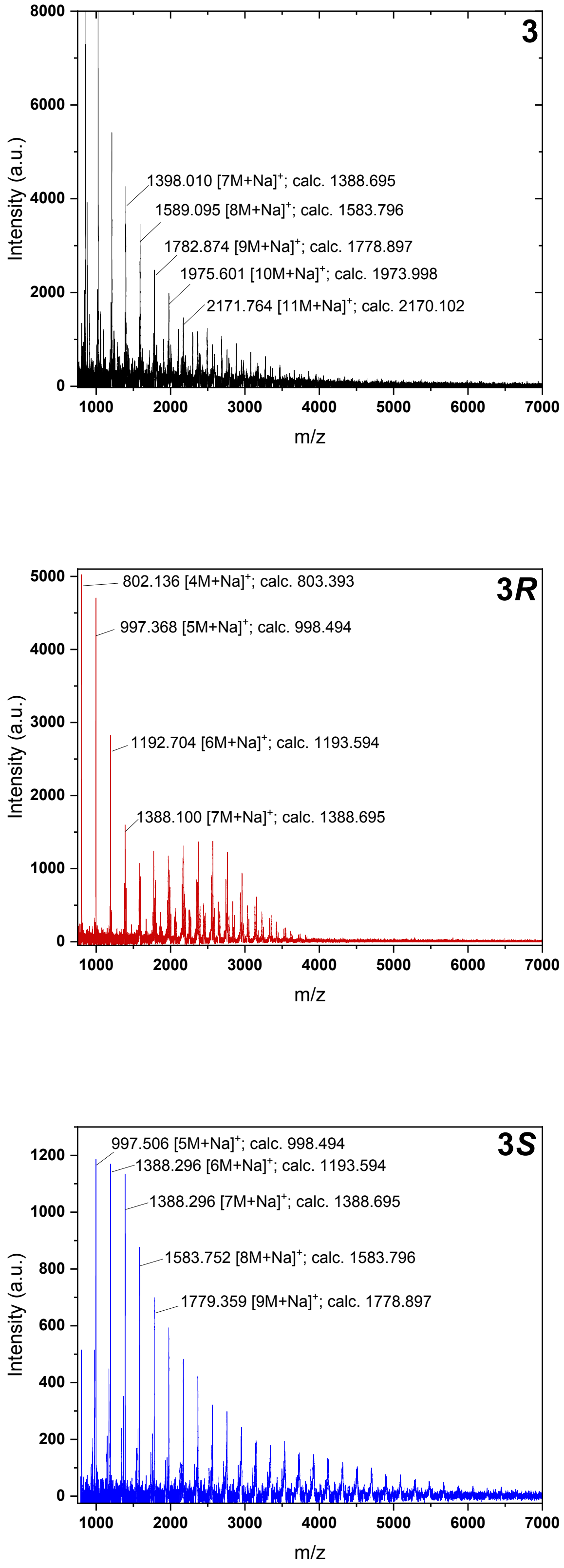

Figure S13: MALDI-ToF spectra of polymers **3**, **3*R*** and **3*S***.

5. NMR spectra

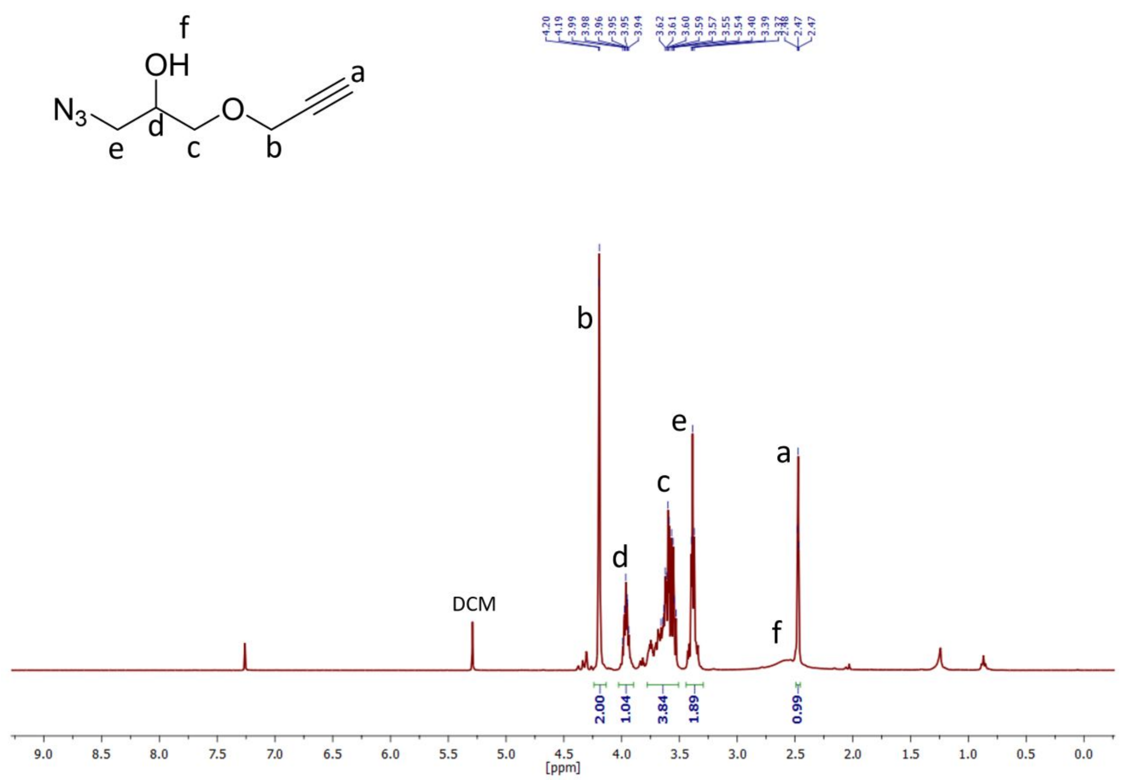

Figure S14: ^{1}H NMR spectrum of **1** (400 MHz, $CDCl_3$).

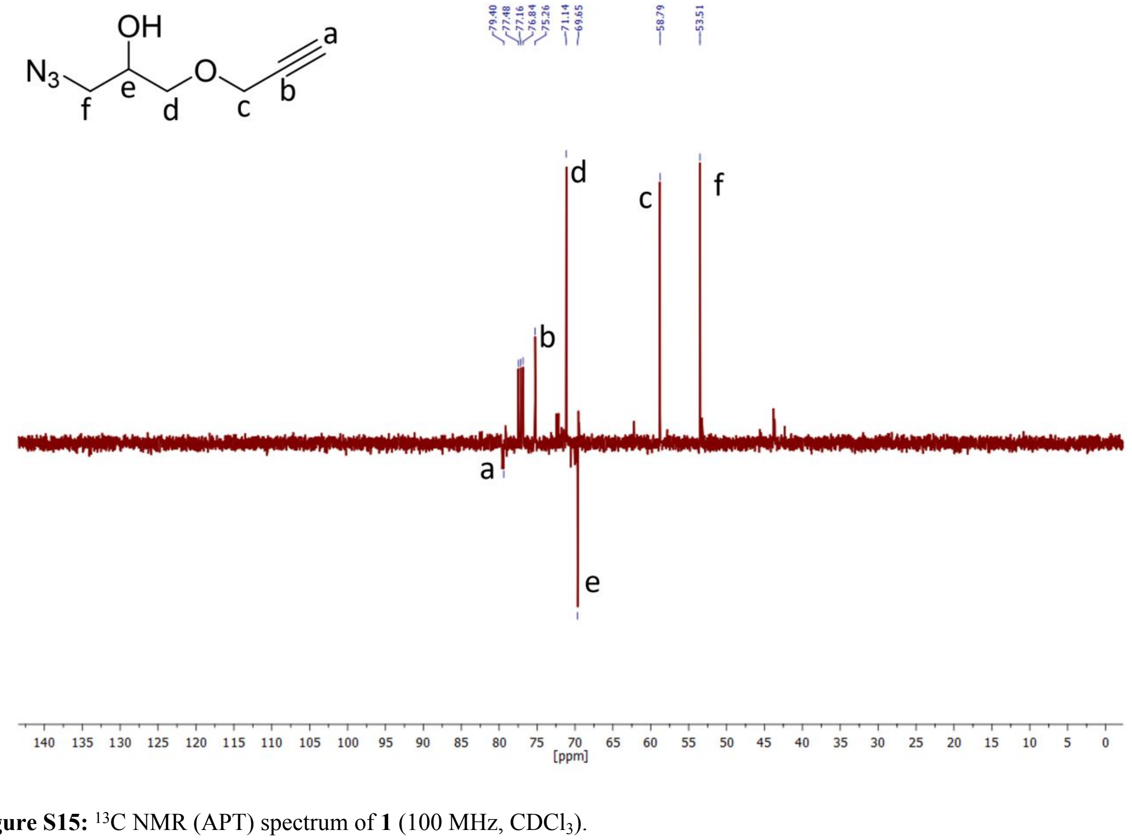

Figure S15: ^{13}C NMR (APT) spectrum of **1** (100 MHz, $CDCl_3$).

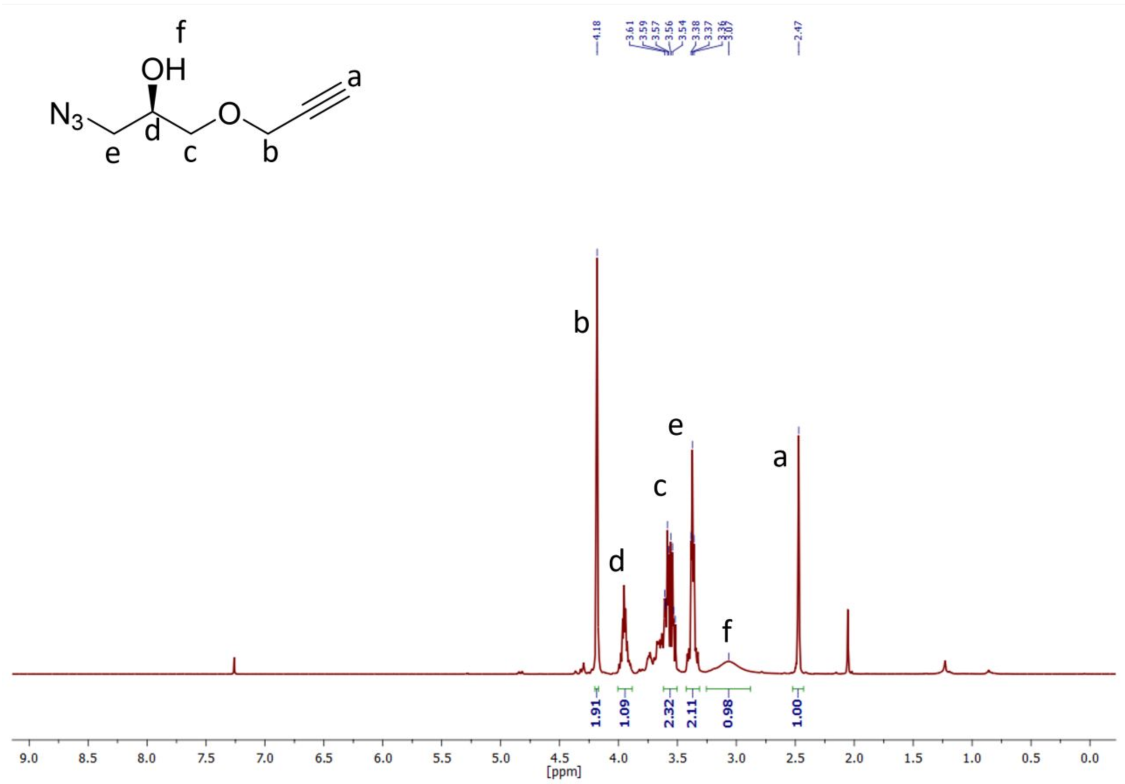

Figure S16: ^{1}H NMR spectrum of **1*R*** (400 MHz, $CDCl_3$).

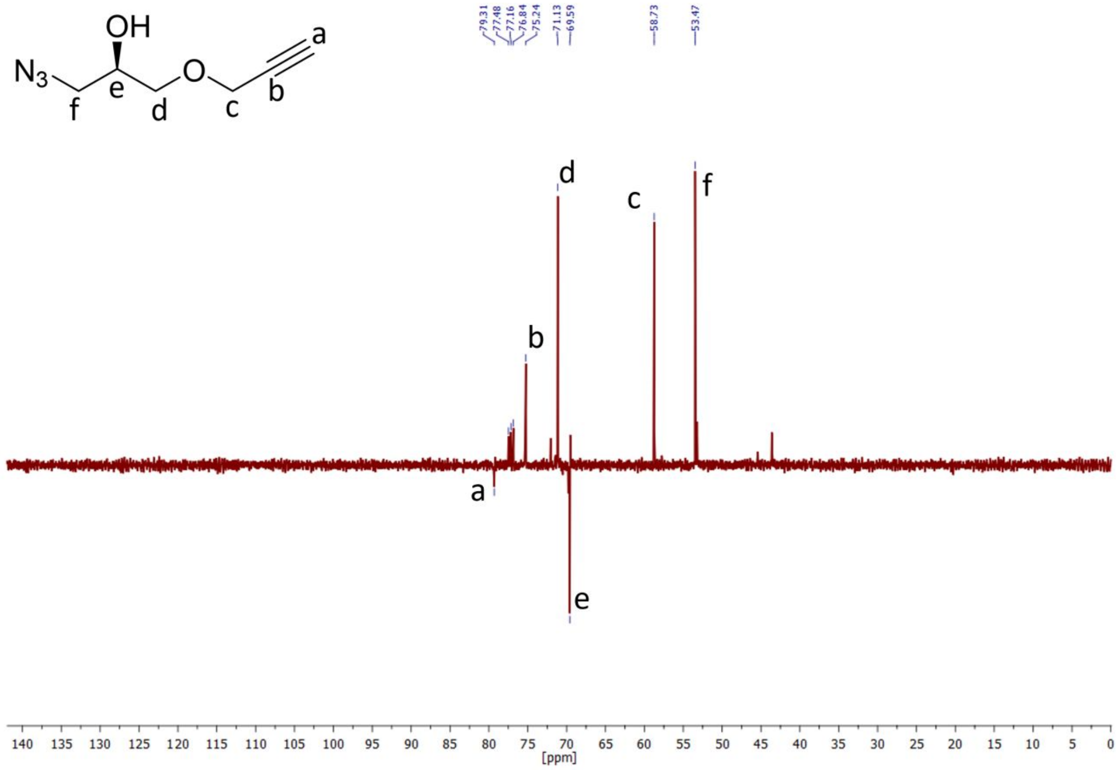

Figure S17: ^{13}C NMR (APT) spectrum of **1*R*** (100 MHz, $CDCl_3$).

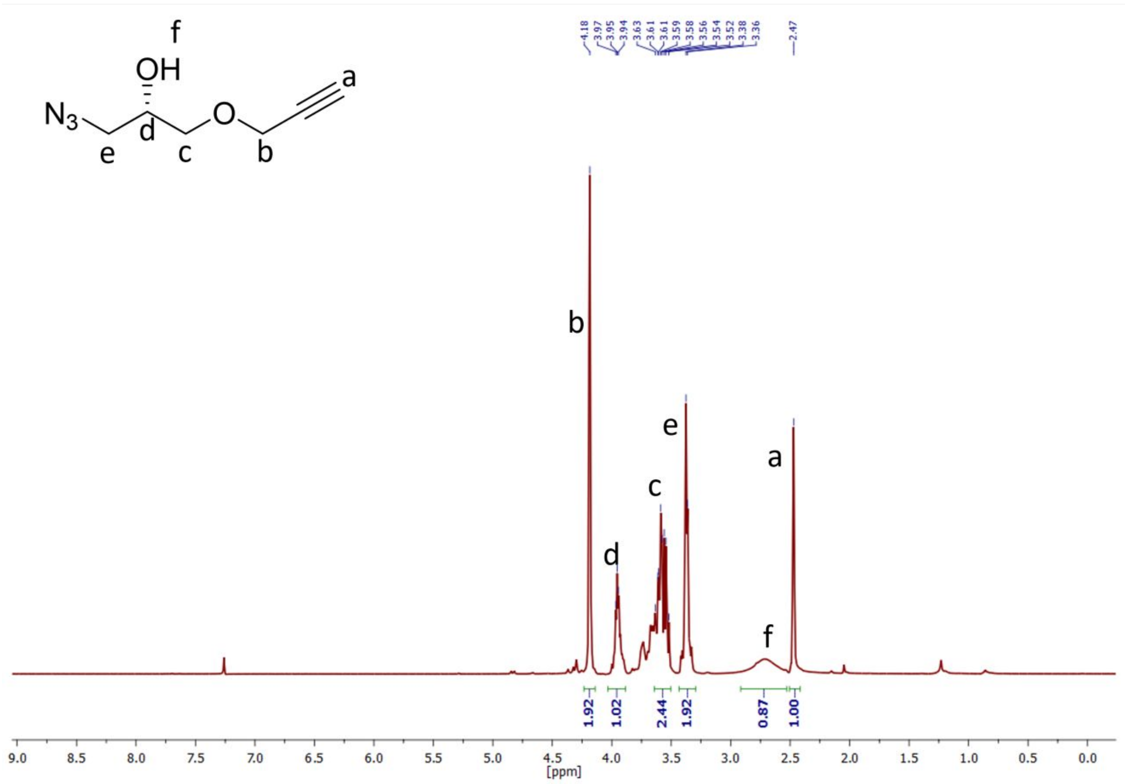

Figure S18: ^{1}H NMR spectrum of **1*S*** (400 MHz, $CDCl_3$).

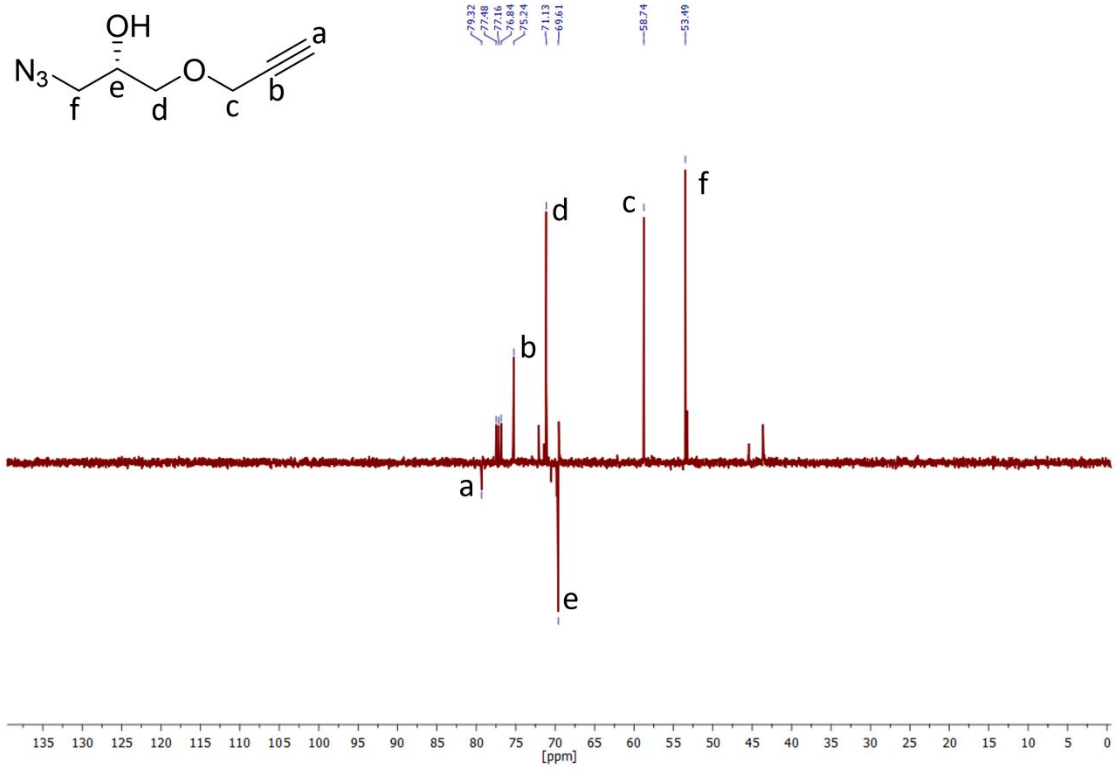

Figure S19: ^{13}C NMR (APT) spectrum of **1*S*** (100 MHz, $CDCl_3$).

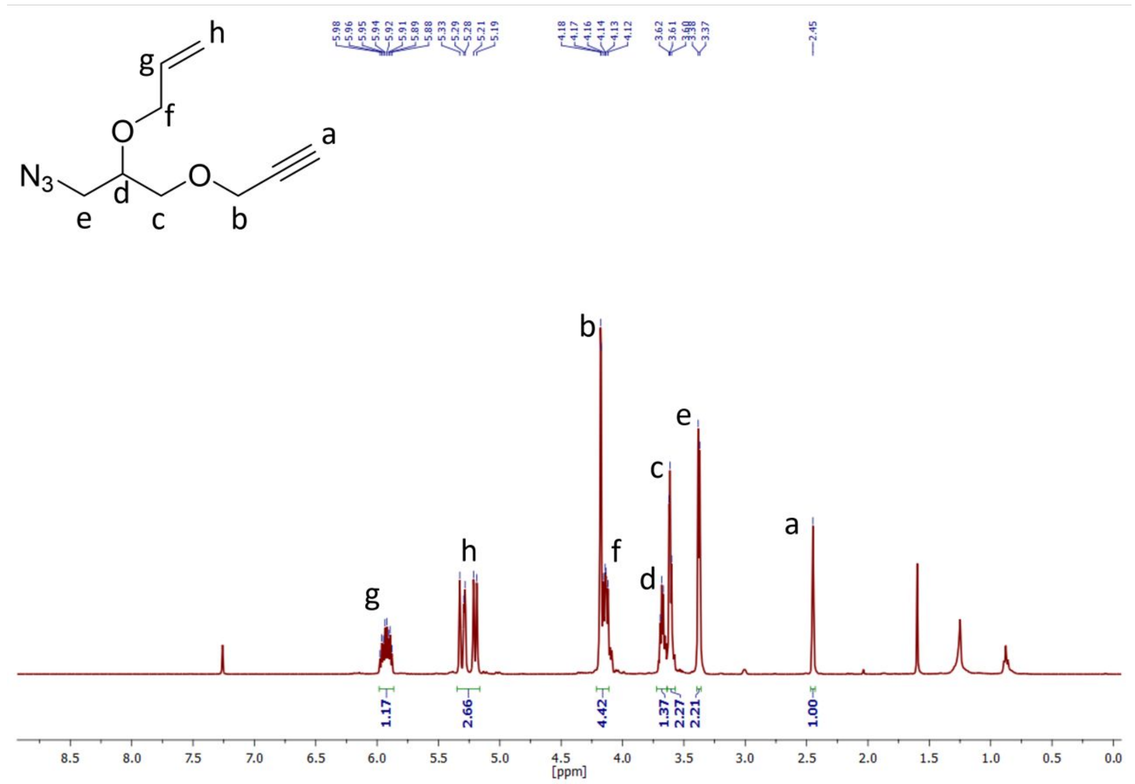

Figure S 20: ^{1}H NMR spectrum of **2** (400 MHz, $CDCl_3$).

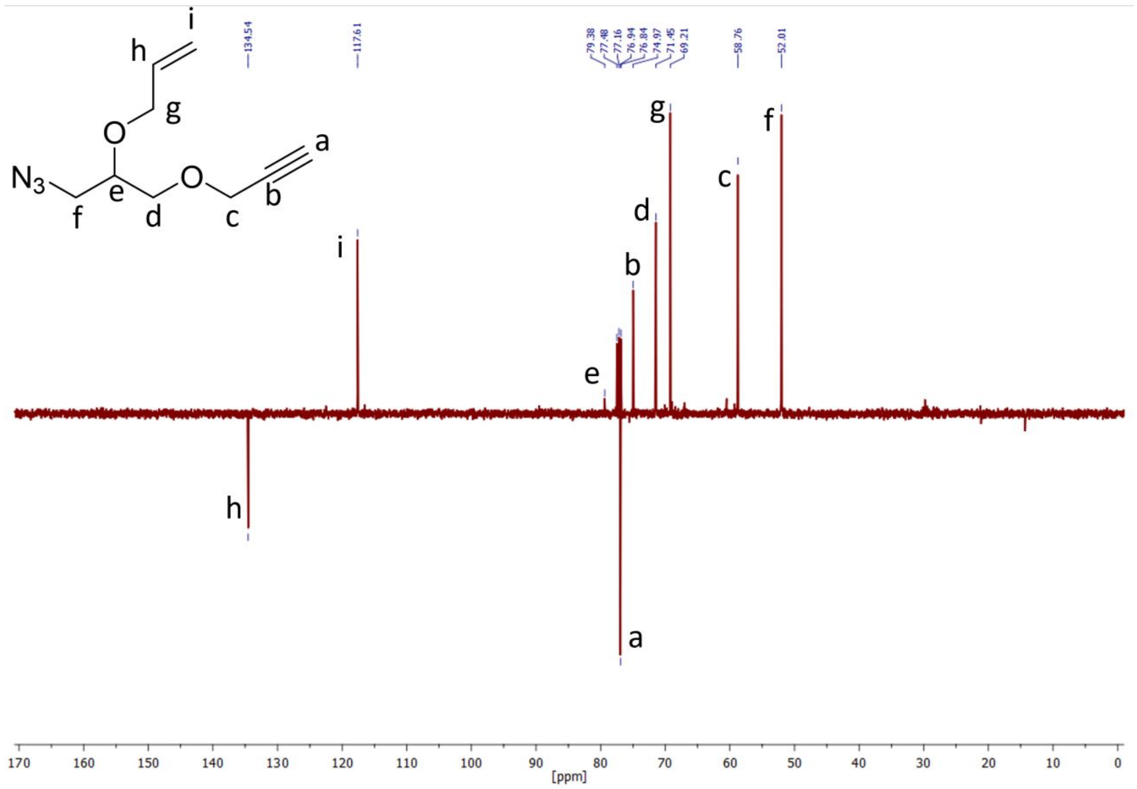

Figure S 21: ^{13}C NMR (APT) spectrum of **2** (100 MHz, $CDCl_3$).

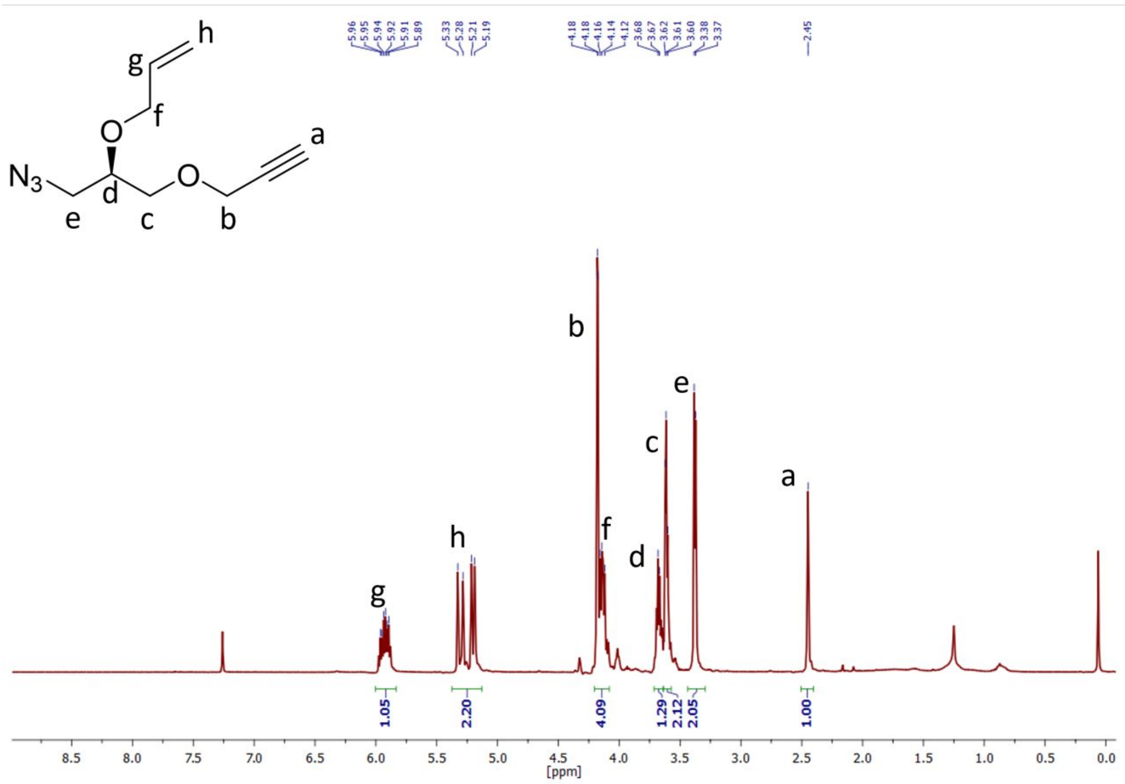

Figure S 22: ^{1}H NMR spectrum of **2*R*** (400 MHz, $CDCl_3$).

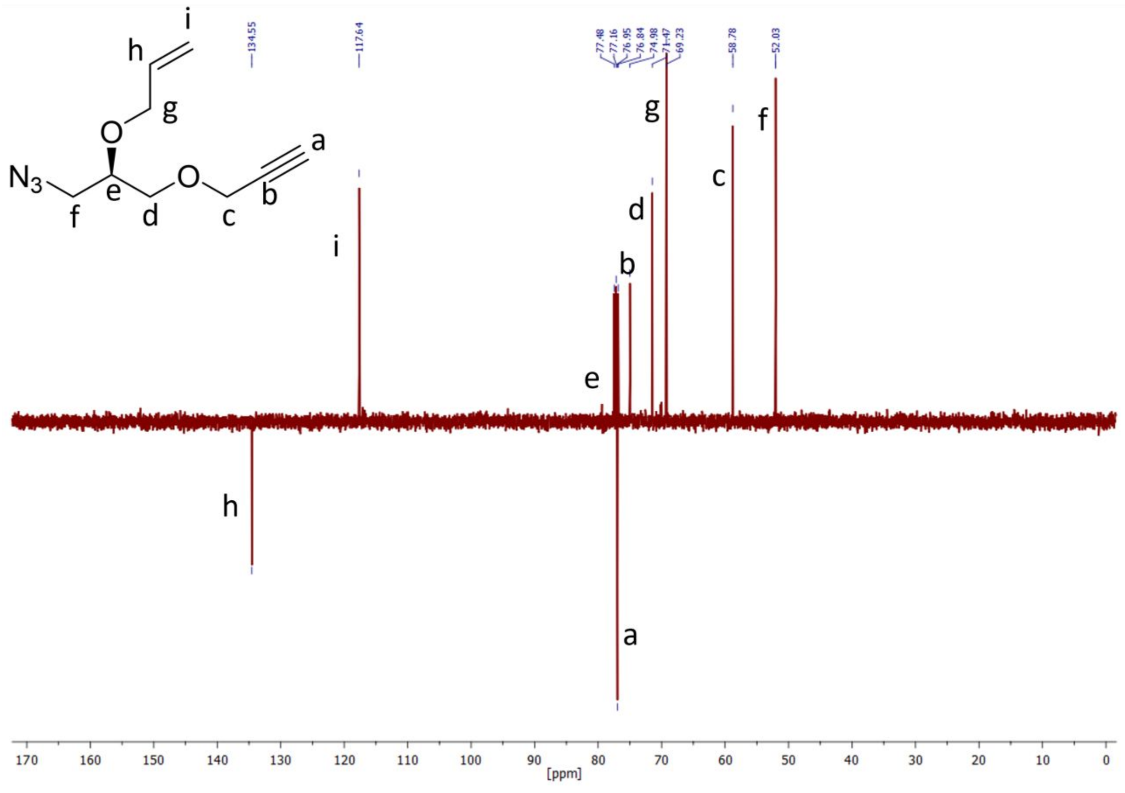

Figure S23: ^{13}C NMR (APT) spectrum of **2*R*** (100 MHz, $CDCl_3$).

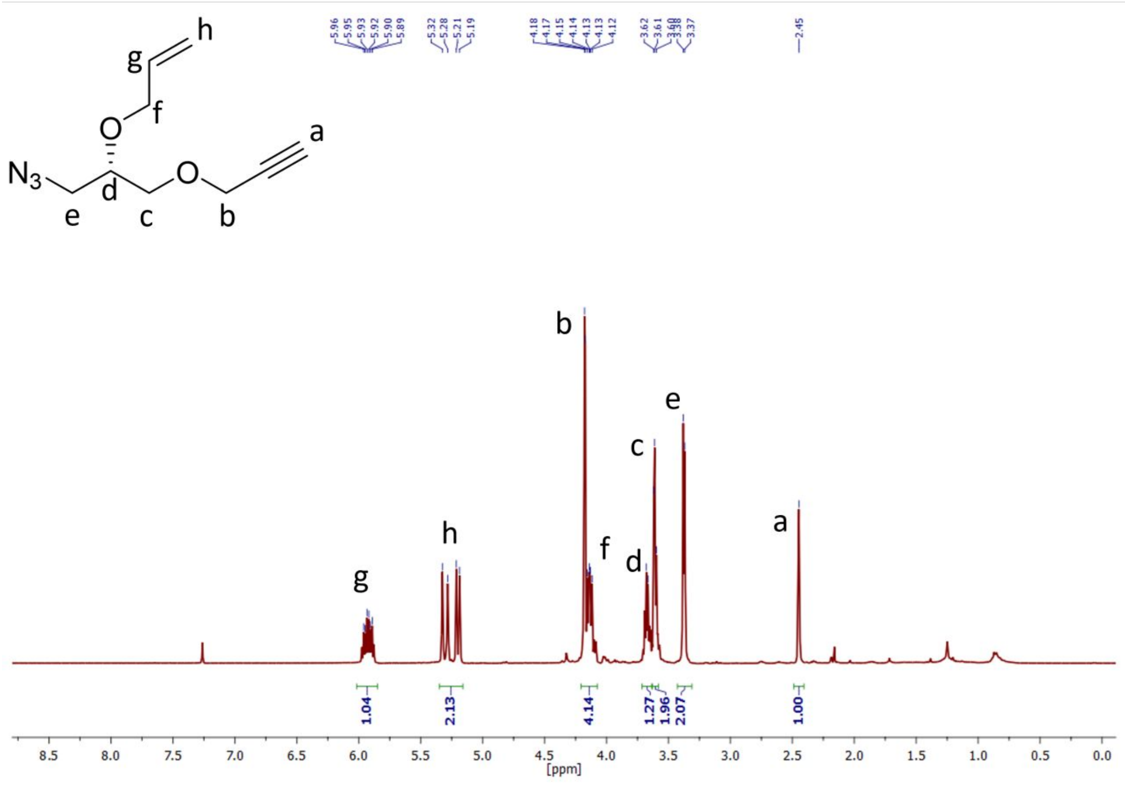

Figure S 24: ^{1}H NMR spectrum of **2*S*** (400 MHz, $CDCl_3$).

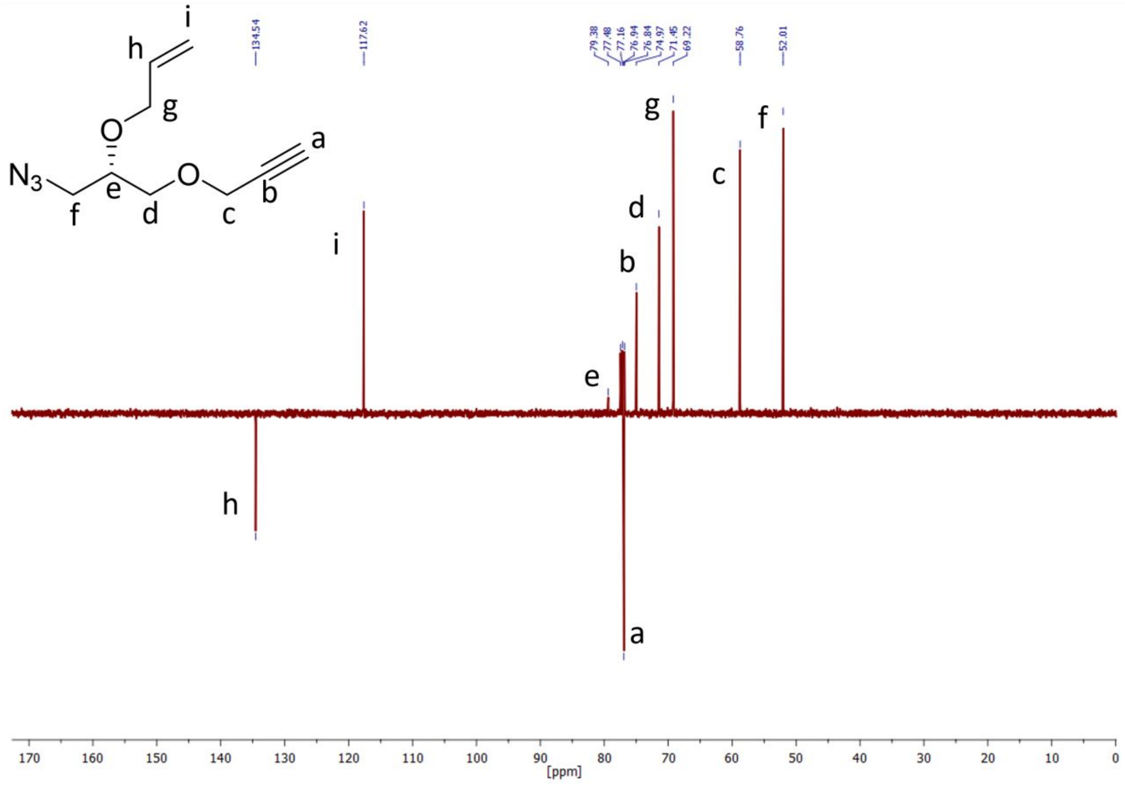

Figure S 25: ^{13}C NMR (APT) spectrum of **2*S*** (100 MHz, $CDCl_3$).

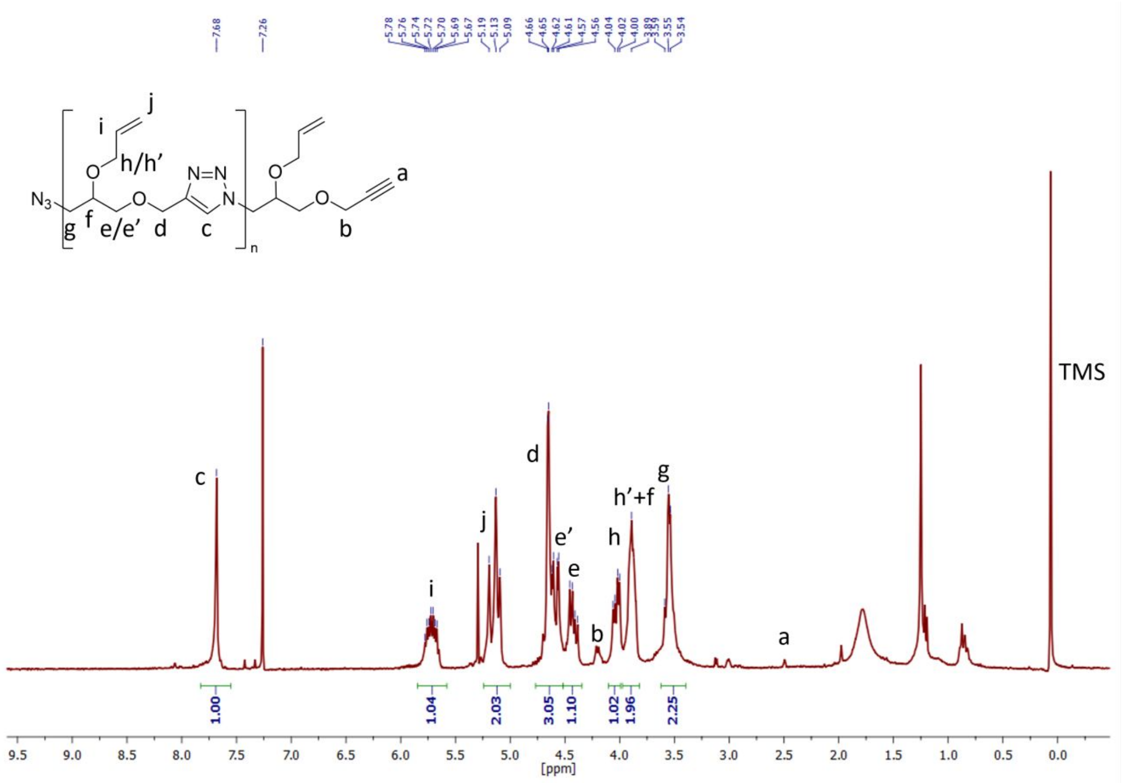

Figure S 26: ^{1}H NMR spectrum of **3** (400 MHz, $CDCl_3$).

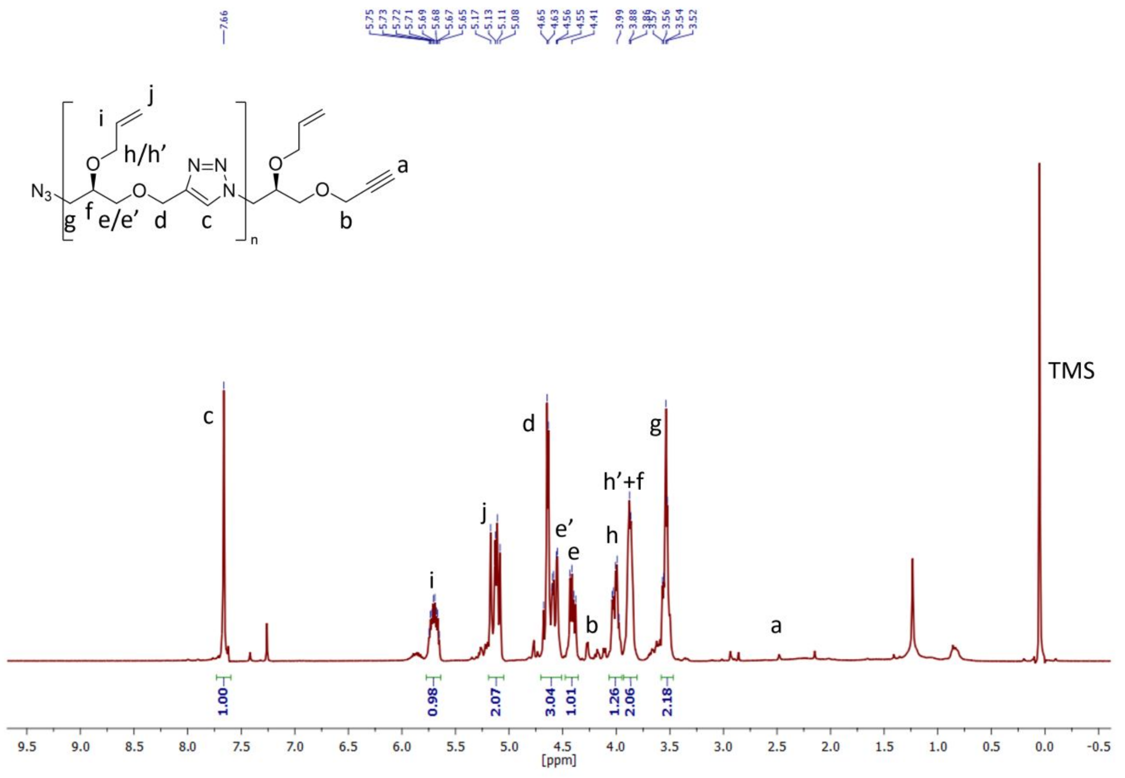

Figure S 27: ^{1}H NMR spectrum of **3*R*** (400 MHz, $CDCl_3$).

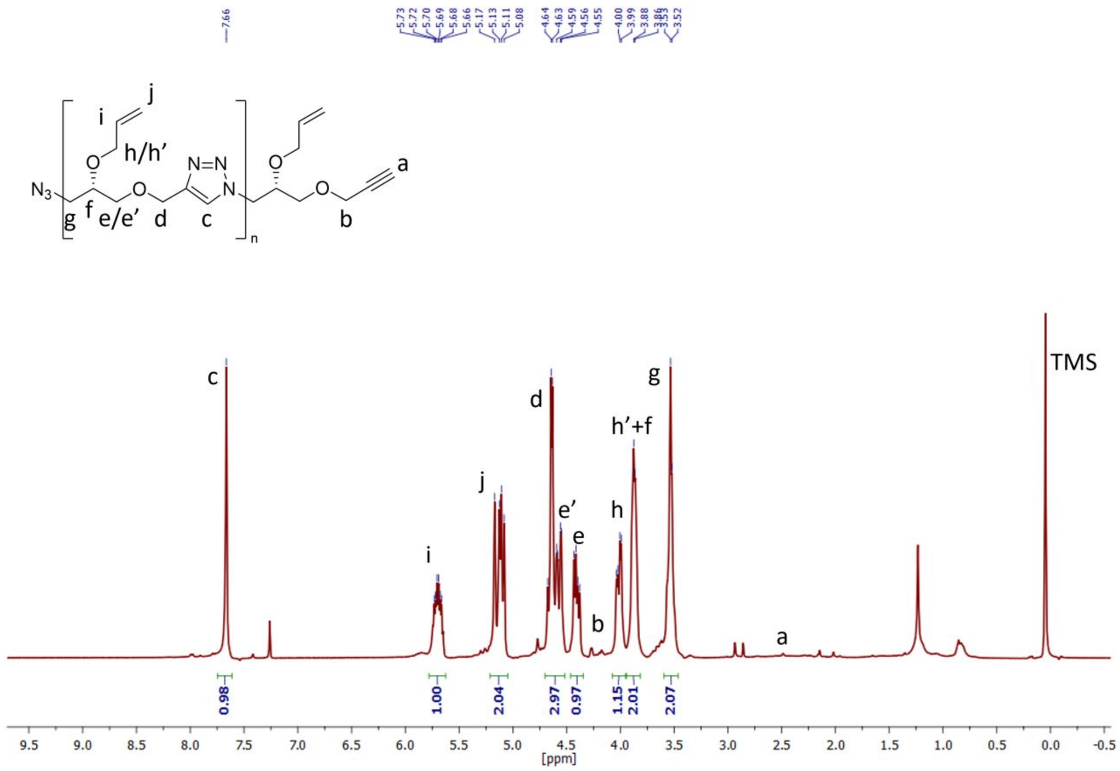

Figure S 28: ^{1}H NMR spectrum of **3*S*** (400 MHz, $CDCl_3$).

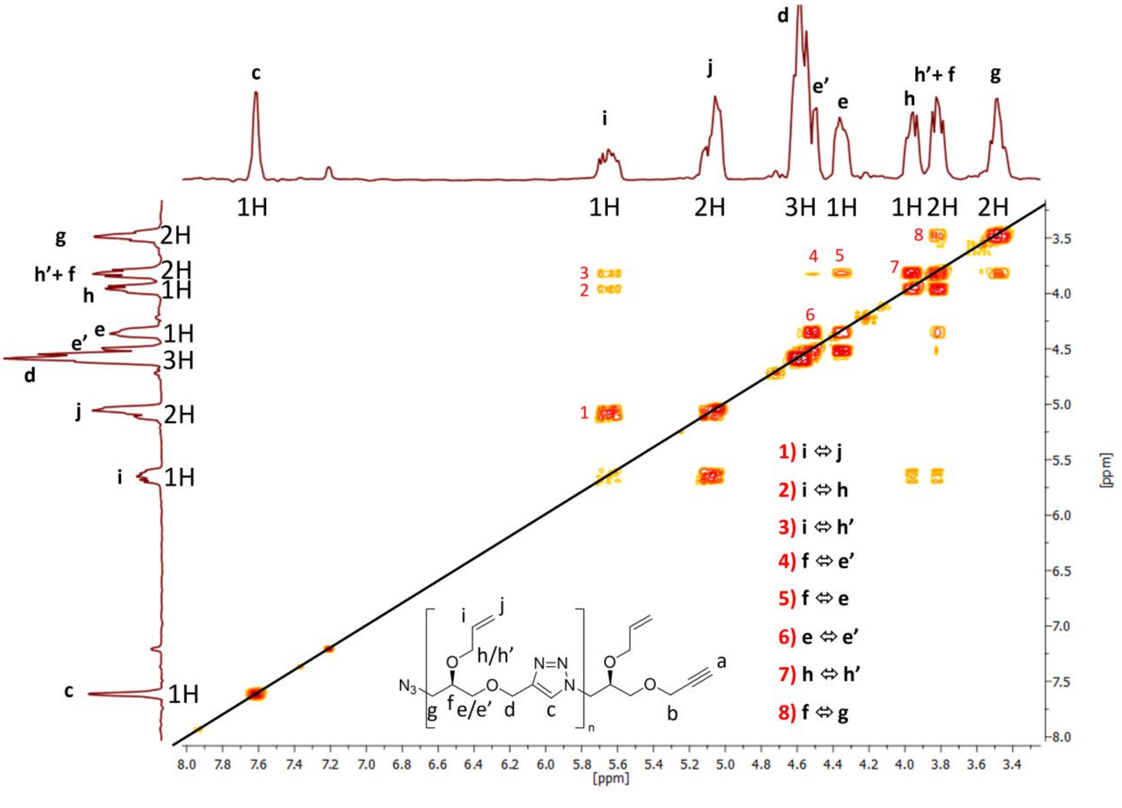

Figure S 29: ^{1}H-^{1}H COSY NMR spectrum of **3*R*** (400 MHz, $CDCl_3$).

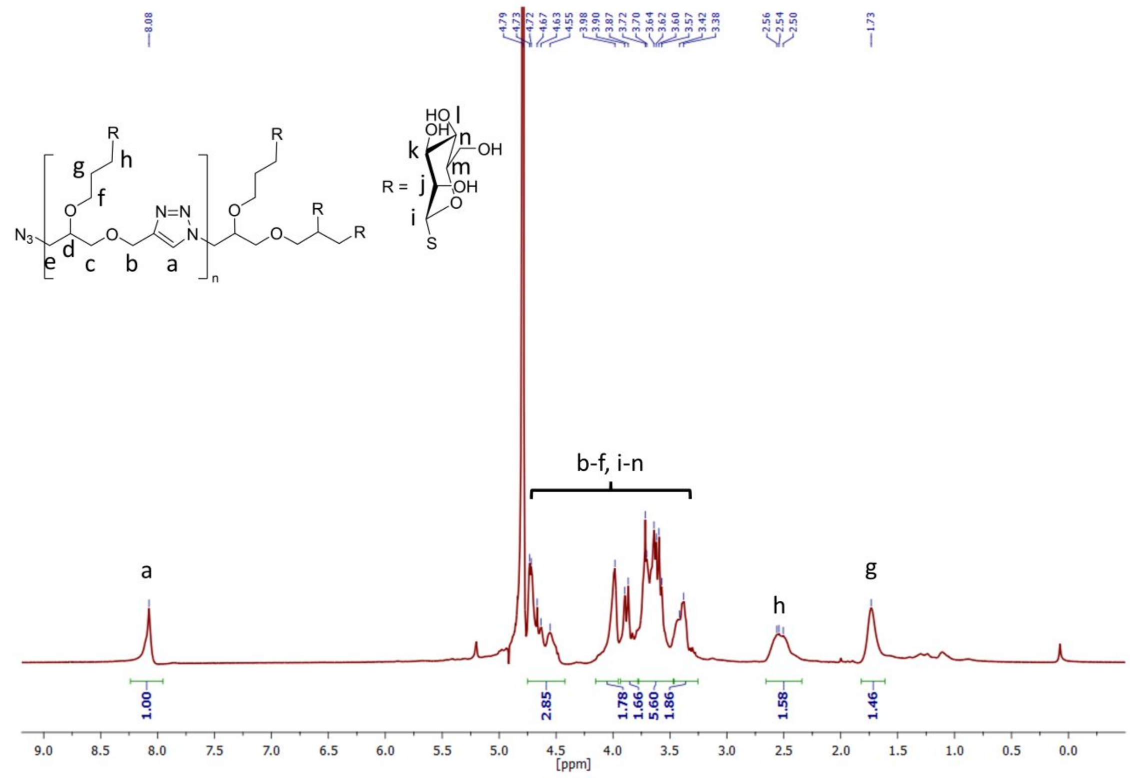

Figure S 30: ^{1}H NMR spectrum of **4** (400 MHz, D_2O).

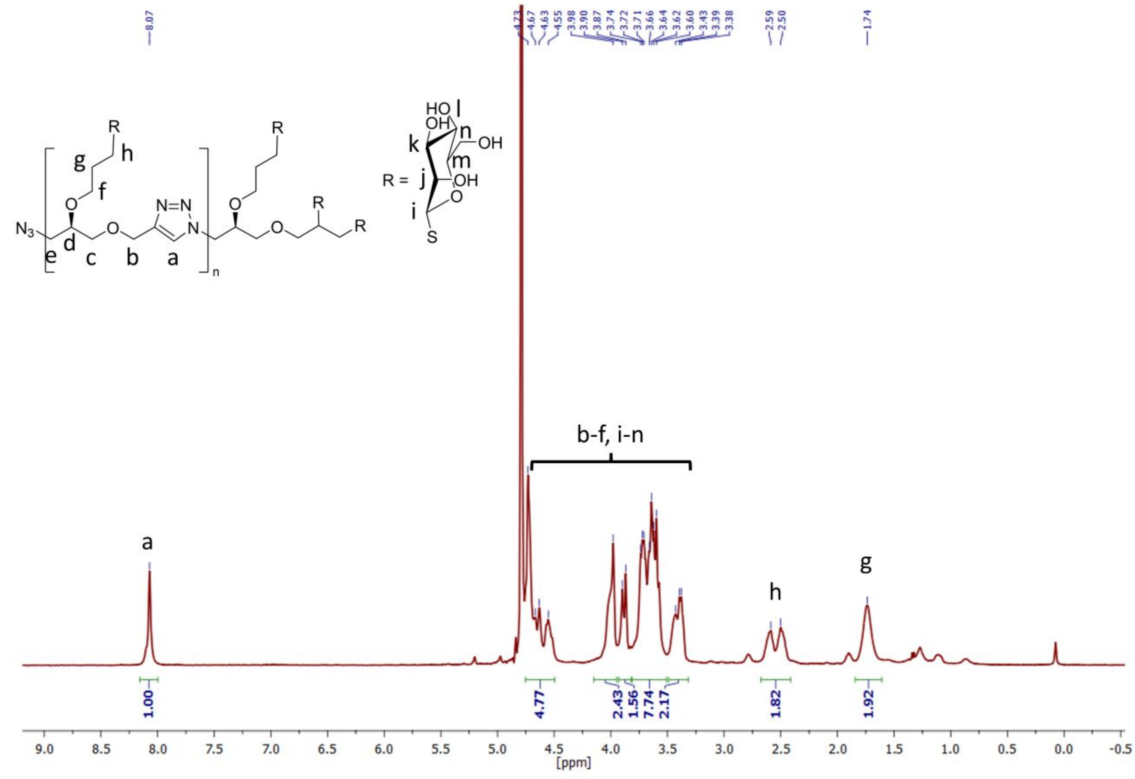

Figure S 31: ^{1}H NMR spectrum of **4*R*** (400 MHz, D_2O).

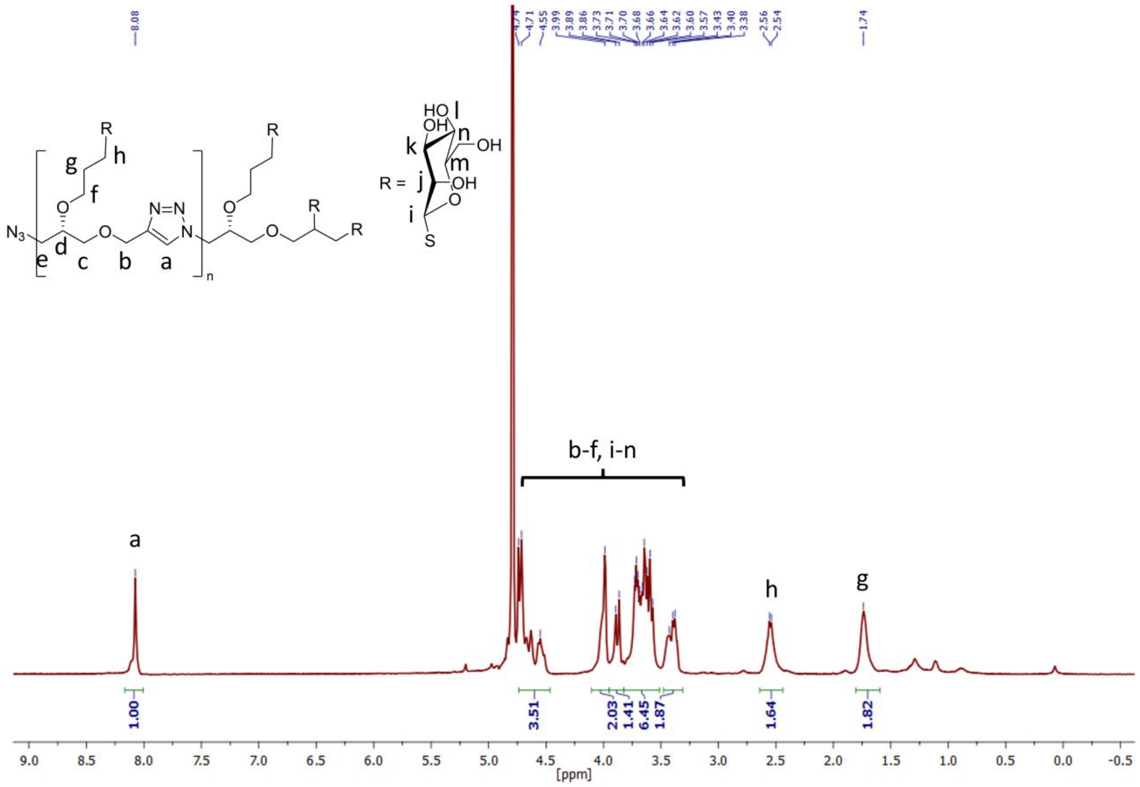

Figure S 32: ^{1}H NMR spectrum of **4*S*** (400 MHz, D_2O).

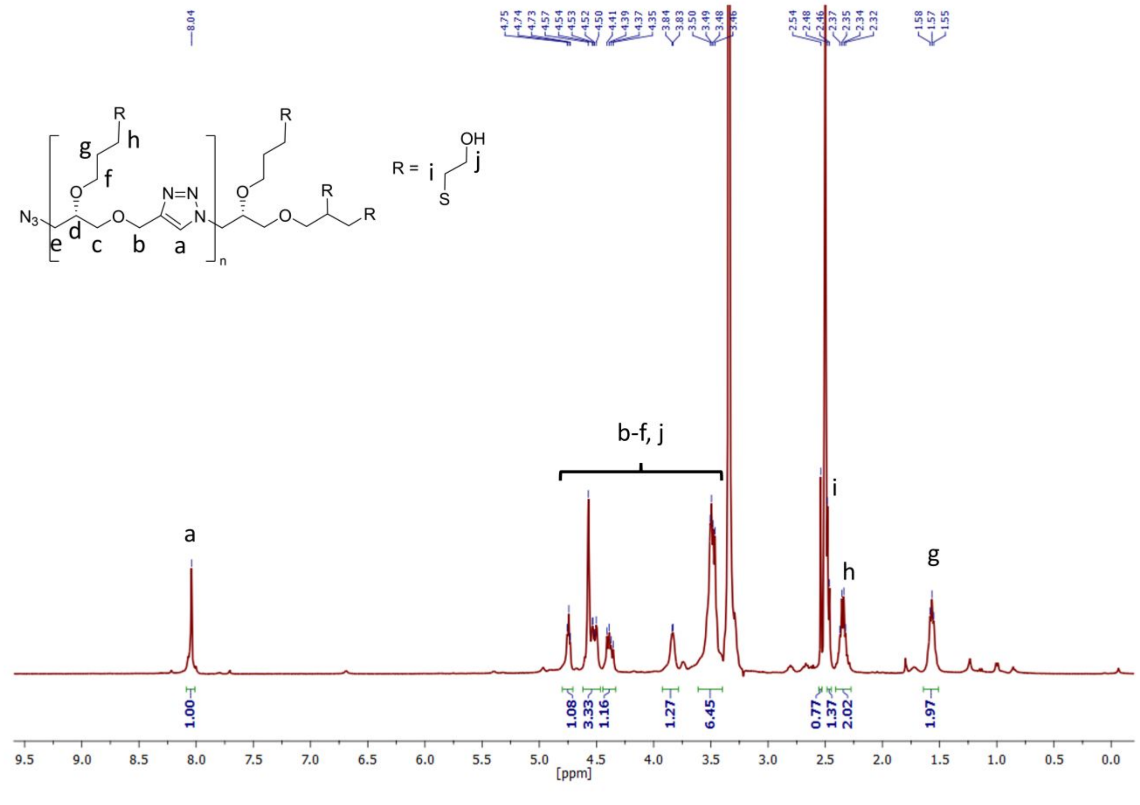

Figure S 33: ^{1}H NMR spectrum of **5** (400 MHz, DMSO-*d*6).

6. SPR data overview

Table 2. Overview of kinetic data (k_a, k_d, R_{max}, K_a, K_d) obtained from fitting of experimental SPR curves with 1:1 Langmuir binding model.

MBL					
Polymer	k_a ($M^{-1}s^{-1}$)	k_d (s^{-1})	R_{max} (RU)	K_a (M^{-1})	K_d (M)
**4**	437	$1.41*10^{-4}$	1150	$3.10*10^{6}$	$3.22*10^{-7}$
**4*R***	1030	$2.29*10^{-6}$	638	$4.50*10^{8}$	$2.22*10^{-9}$
**4*S***	1710	$5.11*10^{-6}$	817	$3.35*10^{8}$	$2.98*10^{-9}$
DC-SIGN					
Polymer	k_a ($M^{-1}s^{-1}$)	k_d (s^{-1})	R_{max} (RU)	K_a (M^{-1})	K_d (M)
**4**	671	$8.83*10^{-4}$	193	$7.60*10^{5}$	$1.32*10^{-6}$
**4*R***	2340	$1.30*10^{-4}$	90	$1.79*10^{7}$	$5.57*10^{-8}$
**4*S***	3200	$4.17*10^{-4}$	154	$7.68*10^{6}$	$1.30*10^{-7}$
DEC-205					
Polymer	k_a ($M^{-1}s^{-1}$)	k_d (s^{-1})	R_{max} (RU)	K_a (M^{-1})	K_d (M)
**4**	1740	$3.41*10^{-3}$	218	$5.10*10^{5}$	$1.96*10^{-6}$
**4*R***	1460	$6.31*10^{-3}$	140	$2.32*10^{5}$	$4.31*10^{-6}$
**4*S***	1690	$5.48*10^{-3}$	151	$3.08*10^{5}$	$3.24*10^{-6}$
CLEC10A					
Polymer	k_a ($M^{-1}s^{-1}$)	k_d (s^{-1})	R_{max} (RU)	K_a (M^{-1})	K_d (M)
**4**	996	$2.47*10^{-3}$	380	$4.04*10^{5}$	$2.48*10^{-6}$
**4*R***	1310	$5.91*10^{-3}$	221	$2.23*10^{5}$	$4.49*10^{-6}$
**4*S***	1310	$4.81*10^{-3}$	255	$2.73*10^{5}$	$3.67*10^{-6}$

7. SPR sensorgrams of polymer 5

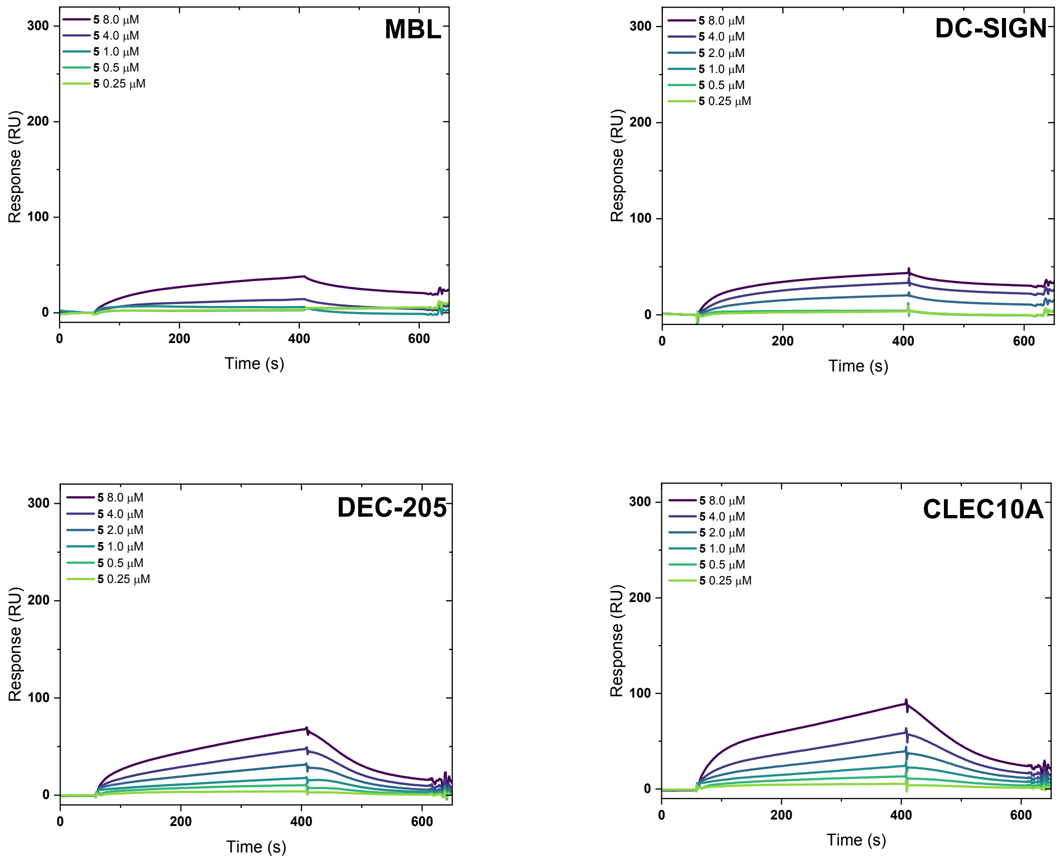

Figure S 34. SPR binding curves of negative control polymer **5** against lectins MBL, DC-SIGN, DEC-205 and CLEC10A**.**

8. IR Analysis of compounds 2, 3 and 4

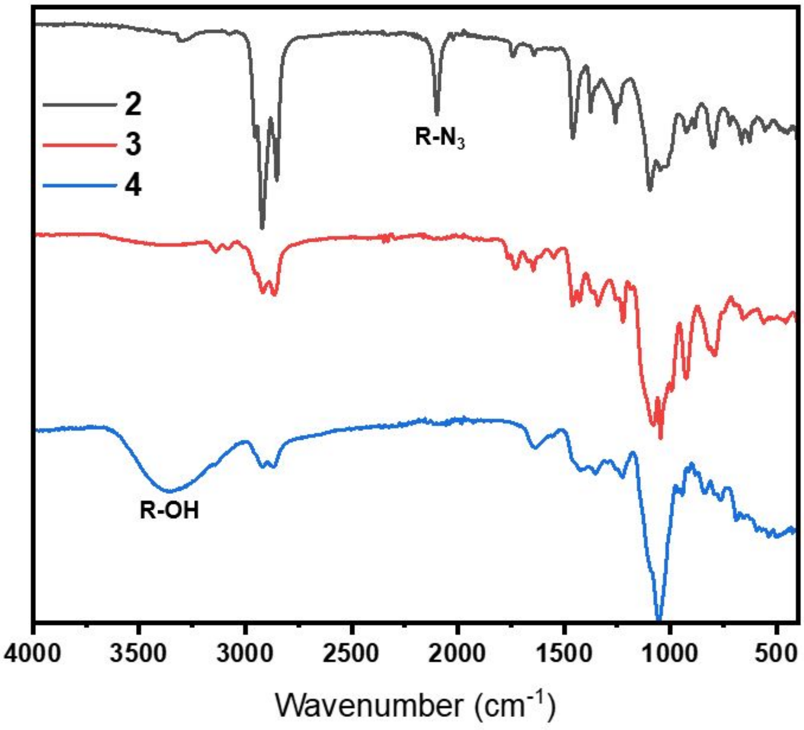

Figure S 35: FTIR spectra of monomer **2** (top), allyl polymer **3** (middle) and glycopolymer **4** (bottom).

9. References

1. Golder, M. R.; Jiang, Y.; Teichen, P. E.; Nguyen, H. V.; Wang, W.; Milos, N.; Freedman, S. A.; Willard, A. P.; Johnson, J. A., Stereochemical Sequence Dictates Unimolecular Diblock Copolymer Assembly. *J. Am. Chem. Soc.* **2018,** *140* (5), 1596-1599.
2. Hartweg, M.; Jiang, Y.; Yilmaz, G.; Jarvis, C. M.; Nguyen, H. V.; Primo, G. A.; Monaco, A.; Beyer, V. P.; Chen, K. K.; Mohapatra, S.; Axelrod, S.; Gomez-Bombarelli, R.; Kiessling, L. L.; Becer, C. R.; Johnson, J. A., Synthetic Glycomacromolecules of Defined Valency, Absolute Configuration, and Topology Distinguish between Human Lectins. *JACS Au* **2021,** *1* (10), 1621-1630.
3. Ishido, Y.; Kanbayashi, N.; Fujii, N.; Okamura, T. A.; Haino, T.; Onitsuka, K., Folding control of a non-natural glycopeptide using saccharide-coded structural information for polypeptides. *Chem. Commun. (Camb.)* **2020,** *56* (18), 2767-2770.
